# LGH-Transformer: A Hybrid CNN–Transformer Model for Multi-Class Bladder Tissue Classification from Cystoscopic Images

**DOI:** 10.3390/ijms27198929

**Published:** 2026-10-08

**Authors:** Hasan Anıl Kurt, Sabire Kılıçarslan, Meliha Merve Çiçekliyurt, Serhat Kiliçarslan

**Affiliations:** 1 Department of Urology, Faculty of Medicine, Çanakkale Onsekiz Mart University, 17100 Çanakkale, Turkey; 2 Department of Medical System Biology, Graduate School of Sciences, Çanakkale Onsekiz Mart University, 17100 Çanakkale, Turkey; 3 Department of Medical Biology, Faculty of Medicine, Çanakkale Onsekiz Mart University, 17100 Çanakkale, Turkey; 4 Department of Software Engineering, Faculty of Engineering, Bandirma Onyedi Eylül University, 10200 Balıkesir, Turkey

**Keywords:** bladder cancer, deep learning, image classification, transfer learning, transformer models

## Abstract

Automated analysis of cystoscopic images may support consistent characterization of bladder tissue patterns, but reported performance is highly sensitive to patient composition, data partitioning, and evaluation methodology. Objective: This study provides an exploratory comparison of convolutional neural networks, Transformer-based models, and a proposed Local–Global Hybrid Transformer (LGH-Transformer) for four-class bladder tissue image classification. The dataset comprised 1754 frames and used the publicly released 1257/308/189 training–validation–held-out test split. The annotation-defined held-out test composition was HGC = 74, LGC = 53, NST = 37, and NTL = 25 (*n* = 189). Images were resized to 224 × 224 pixels, normalized, and augmented only in the training subset. Adam and RMSprop were evaluated. Accuracy and macro-averaged precision, recall, and F1-score were calculated from the integer-count evaluation matrices. The highest held-out accuracy, 187/189 (0.9894), was reached by seven model–optimizer configurations: DenseNet201–RMSprop, XCiT Medium 24 P16–Adam, ConvNeXt Base–Adam, CoAtNet–Adam, ConvNeXt Base–RMSprop, CoAtNet–RMSprop, and the proposed LGH-Transformer–RMSprop; MobileNet–Adam and Xcep-tion–RMSprop each yielded 185/189 (0.9788), and LGH-Transformer–Adam yielded 186/189 (0.9841). On a 189-frame test set, differences in one to two frames do not establish statistical superiority, so these configurations are reported as a top-accuracy tier rather than as a ranked superiority claim. Within this tier, the LGH-Transformer had the smallest computational footprint (≈18.2 M parameters and ≈4.1 GFLOPs, versus 20.0–88.6 M parameters and 4.3–16.2 GFLOPs for the other top-tier models). Component-wise ablation experiments showed accuracy decreases of 0.0106, 0.0053, and 0.0053 after removing the CNN tokenizer, local attention, and global attention, respectively. The LGH-Transformer reaches the top accuracy tier of this 18-architecture benchmark at the lowest parameter and FLOP budget within that tier, supporting an internally consistent, efficiency-oriented comparison within the available cohort. The absence of task-aligned external validation and the small number of held-out patients require the findings to be interpreted as exploratory internal validation rather than evidence of clinical generalizability.

## 1. Introduction

Bladder cancer is a significant urological malignancy, characterized by high morbidity and mortality rates worldwide [1]. The most common histopathological type of disease is urothelial (transitional cell) carcinoma. In clinical practice, the disease is divided into three main groups: non-muscle invasive (NMIBC), muscle invasive, and metastatic [2]. Cystoscopy is the gold standard for evaluation and the diagnosis is confirmed by pathological assessment of a biopsy and transurethral resection of a bladder tumor (TURBT) [3,4].

Although cystoscopy provides a direct, real-time view of the bladder mucosa, adequate TURBT sampling, including detrusor muscle when appropriate, is essential for precise risk classification and treatment planning [4].

The diagnostic performance of cystoscopy is not uniform across settings and lesion types. In adults presenting with hematuria, an EAU Guidelines Office systematic review reported sensitivity ranging from 87% to 100% and specificity from 64% to 100% across studies [5]. Across studies, the sensitivity of conventional white-light cystoscopy (WLC) varied from 47% to 100% [5].

Even during surveillance, clinically relevant lesions can be missed at the index examination. Among 181 initially negative cystoscopies with follow-up examinations, 17 (9.4%) were subsequently identified as false-negative cases after retrospective video review. The overall sensitivity and specificity were 81% and 73%, respectively [6].

Flat lesions such as carcinoma in situ (CIS) are often missed with WLC. Enhanced visualization techniques, including blue light cystoscopy (BLC) and narrow band imaging (NBI), can improve sensitivity, especially for flat disease [7,8], but false positives may increase [7]. BLC has been associated with reduced recurrence and improved recurrence-free survival compared with WLC in non-muscle invasive bladder cancer (NMIBC) [7,9].

Against this clinical background, computer-aided image analysis has clear appeal. Deep learning models can learn discriminative visual patterns from large image collections and may help standardize interpretation under real-world cystoscopy artifacts such as variable illumination, specular reflections, motion blur, and viewpoint changes.

Transfer learning enables models pretrained on large natural-image datasets to be adapted to medical imaging tasks and is particularly useful when only limited annotated cystoscopic data are available [10].

Vision Transformers (ViTs) extend self-attention to image classification by representing an image as a sequence of patches. This allows the model to capture relationships between distant image regions, which may be useful when local appearance alone is insufficient for classification [11]. ViT-based approaches have already been studied in bladder cancer. Khedr et al. evaluated Vision Transformers for bladder cancer classification using histopathological images, while Kurata et al. investigated a ViT-based model for identifying muscle-invasive bladder cancer on MRI [12,13]. These studies show that attention-based models can be applied to different bladder cancer imaging modalities. However, their tasks, image types, class definitions, and validation procedures differ from cystoscopic frame classification, so their reported performance cannot be directly transferred to the present setting.

Deep learning has also been applied directly to cystoscopic images. Ali et al. used pretrained CNNs for blue-light cystoscopy during transurethral resection of bladder tumors and reported strong diagnostic performance for both malignancy detection and assessment of tumor invasiveness [14]. In another endoscopic bladder tissue study, Devi et al. proposed a seventeen-layer deep convolutional neural network and compared it with established CNN backbones such as DenseNet, ResNet, VGG, and Inception [15]. These studies support the use of convolutional feature extraction for cystoscopic tissue recognition. At the same time, they also illustrate an important limitation of the existing literature: model performance is often reported under different data partitions and training procedures, which makes architecture-to-architecture comparison difficult.

Hybrid CNN-Transformer approaches have been introduced to combine the local inductive bias of convolution with the long-range modeling ability of self-attention. Amaouche et al. investigated such a hybrid design in cystoscopic bladder cancer imaging, using convolutional layers for local feature extraction and Transformer-based attention for global context [16]. Evidence from other endoscopic applications also supports this design direction. Kusters et al. compared CNNs and Transformers across gastrointestinal endoscopic image-analysis tasks and found that both model families can be competitive depending on the task and training setting [17]. Chou et al. used a hybrid EfficientNet-ViT framework for esophageal endoscopic detection and reported high classification performance [18]. Although these studies involve different anatomical sites and clinical endpoints, they provide a useful basis for examining whether local and global feature modeling can be complementary in endoscopic images.

For the proposed LGH-Transformer, the specific motivation for placing a CNN tokenizer before self-attention is to preserve local mucosal texture and boundary cues at the point where image pixels are converted into tokens. The tokenizer is intended to provide the attention layers with compact, locality-aware representations rather than requiring self-attention to learn these short-range visual structures directly from patch tokens. The subsequent local- and global-attention stages can then combine fine tissue appearance with broader spatial context, which is relevant when visually similar bladder tissue classes differ in both local surface patterns and their surrounding structural organization.

The broader medical-imaging literature provides a similar picture. Atabansi et al. reviewed Transformer applications in histopathological image analysis and highlighted their ability to model global context and complex spatial patterns, while also noting their computational cost and dependence on sufficiently large annotated datasets [11]. Hybrid designs have therefore been explored as a practical alternative because they can combine convolutional locality with attention-based contextual modeling. Although evidence from histopathology does not provide a direct benchmark for bladder cystoscopy, it supports the rationale for comparing pure attention models with architectures that retain convolutional inductive bias and with lightweight models designed for lower computational cost.

Taken together, previous studies show that CNNs, Vision Transformers, and hybrid architectures can all achieve strong results in medical and endoscopic image classification. However, the literature remains difficult to compare because studies differ in imaging modality, class definitions, model pretraining, optimization, data augmentation, and validation strategy. This is particularly important for cystoscopic datasets, where many frames may originate from the same patient. Image-level splitting can therefore produce optimistic estimates if correlated frames from one patient appear in both training and evaluation sets. In addition, few bladder cystoscopy studies have compared lightweight CNNs, pure Transformers, hierarchical attention models, and hybrid architectures under the same patient-wise split and optimizer settings. This methodological gap motivated the structured comparison used in the present study.

Accordingly, this study compares representative CNN, Transformer, and hybrid architectures, including the proposed LGH-Transformer, under a common patient-wise evaluation framework. The selected models span lightweight and larger backbones, pure and hierarchical attention designs, and architectures that combine convolution with attention.

The main contributions of this study are:

- A comparison of CNN, Transformer, hybrid, and modern vision models using the same patient-disjoint public data partitions.
- A comparison of Adam and RMSprop within the evaluated architectures.
- Development of the LGH-Transformer, which combines convolutional tokenization with local and global attention.
- Descriptive evaluation using overall and class-specific metrics, with particular attention to HGC detection.
- Comparison of predictive performance with parameter count and FLOPs.

These analyses provide an internally consistent comparison within the available cohort. They are not intended to establish clinical generalizability, which requires validation in larger independent patient datasets.

## 2. Results

**Figure 1:**
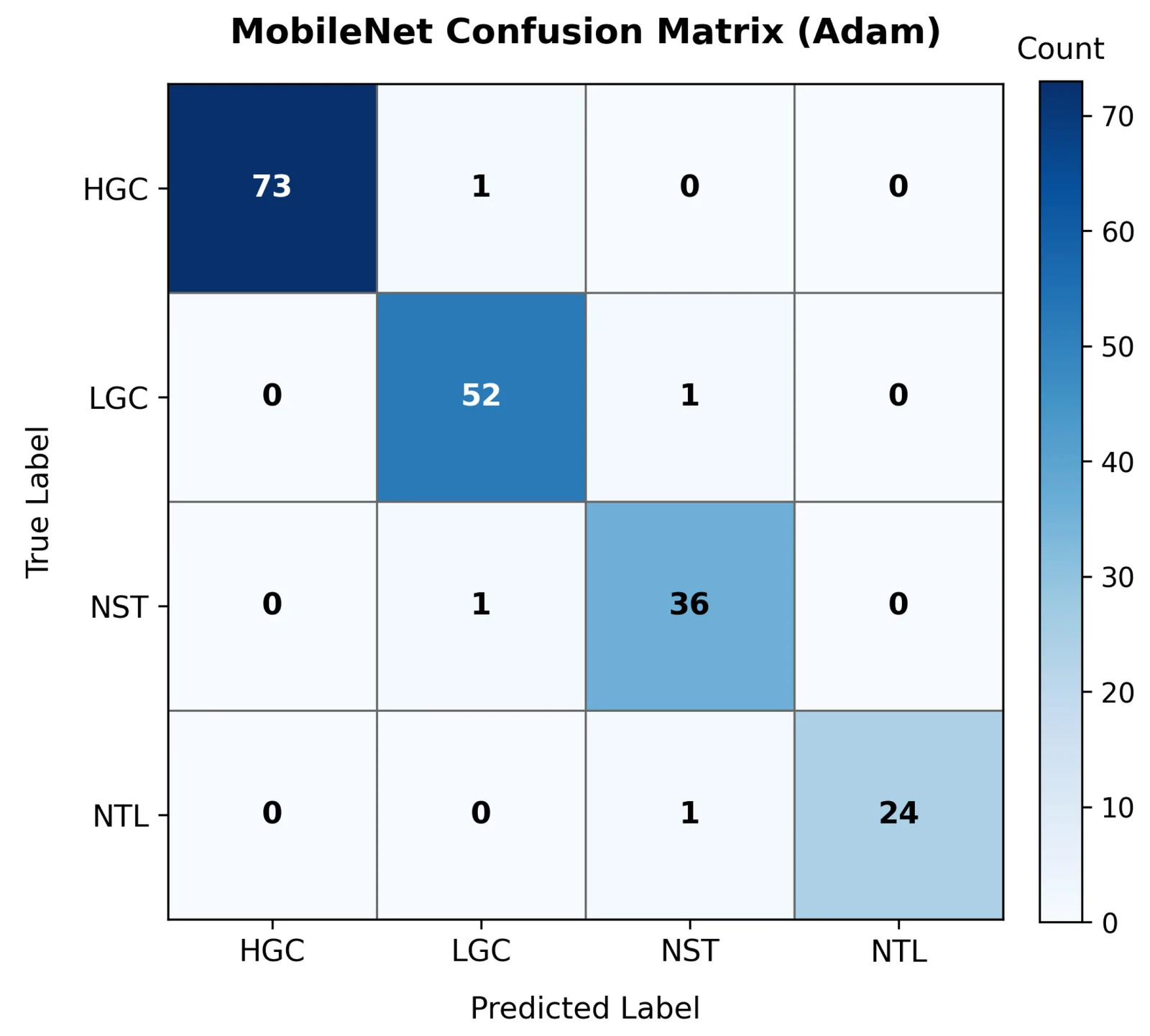
MobileNet confusion matrix (Adam).

**Figure 2:**
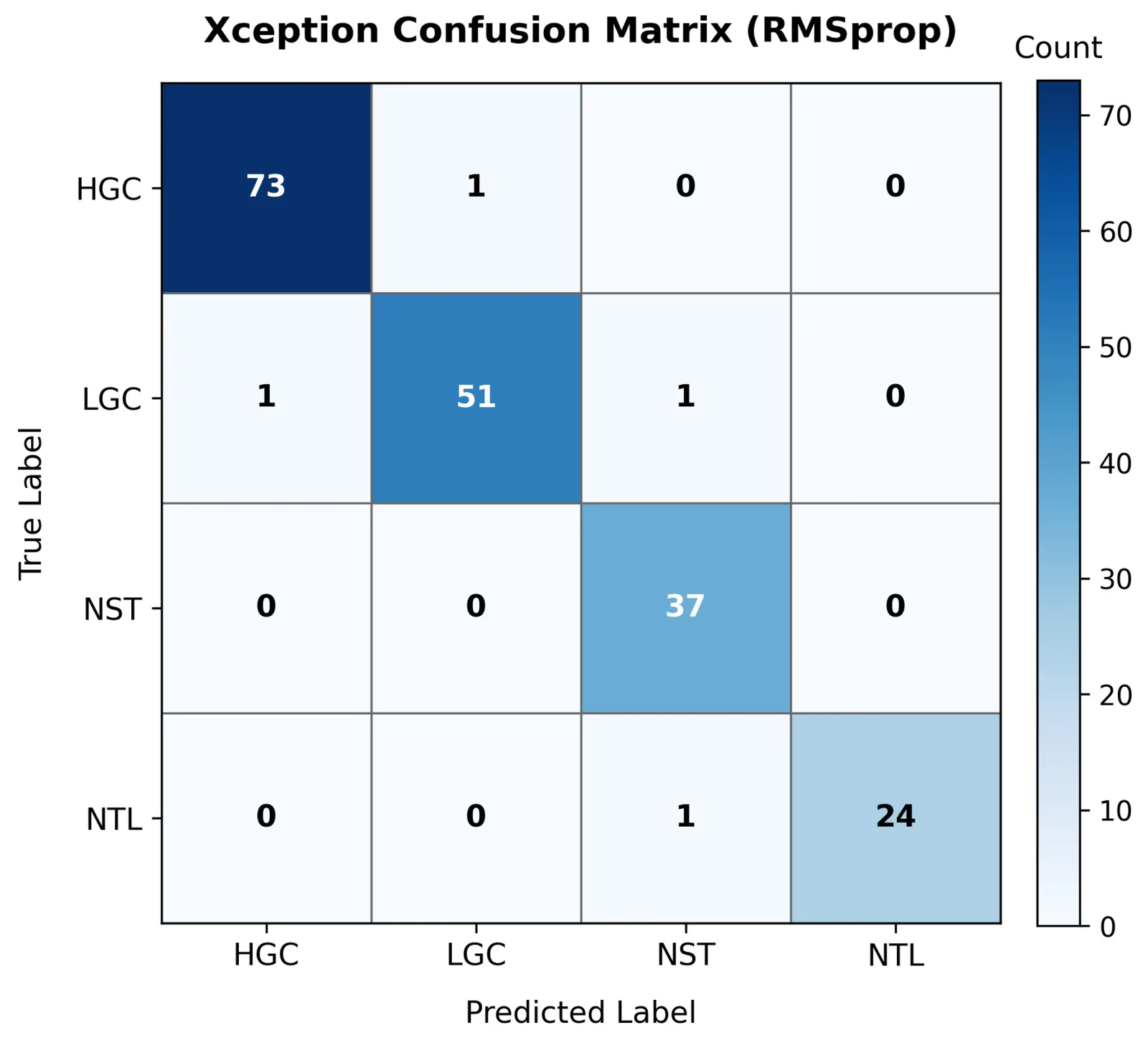
Xception confusion matrix (RMSprop).

**Figure 3:**
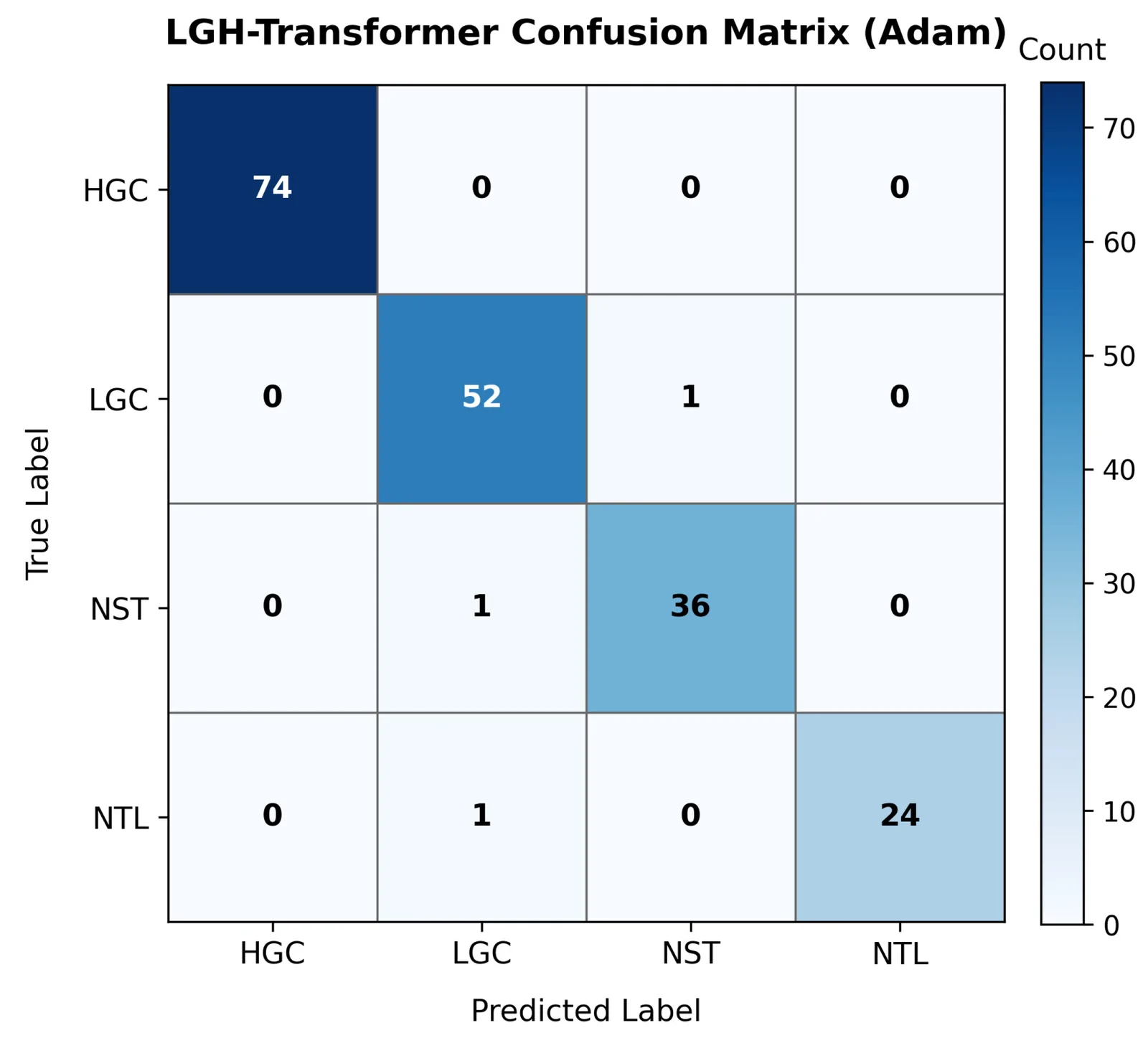
Proposed LGH-Transformer confusion matrix (Adam).

**Figure 4:**
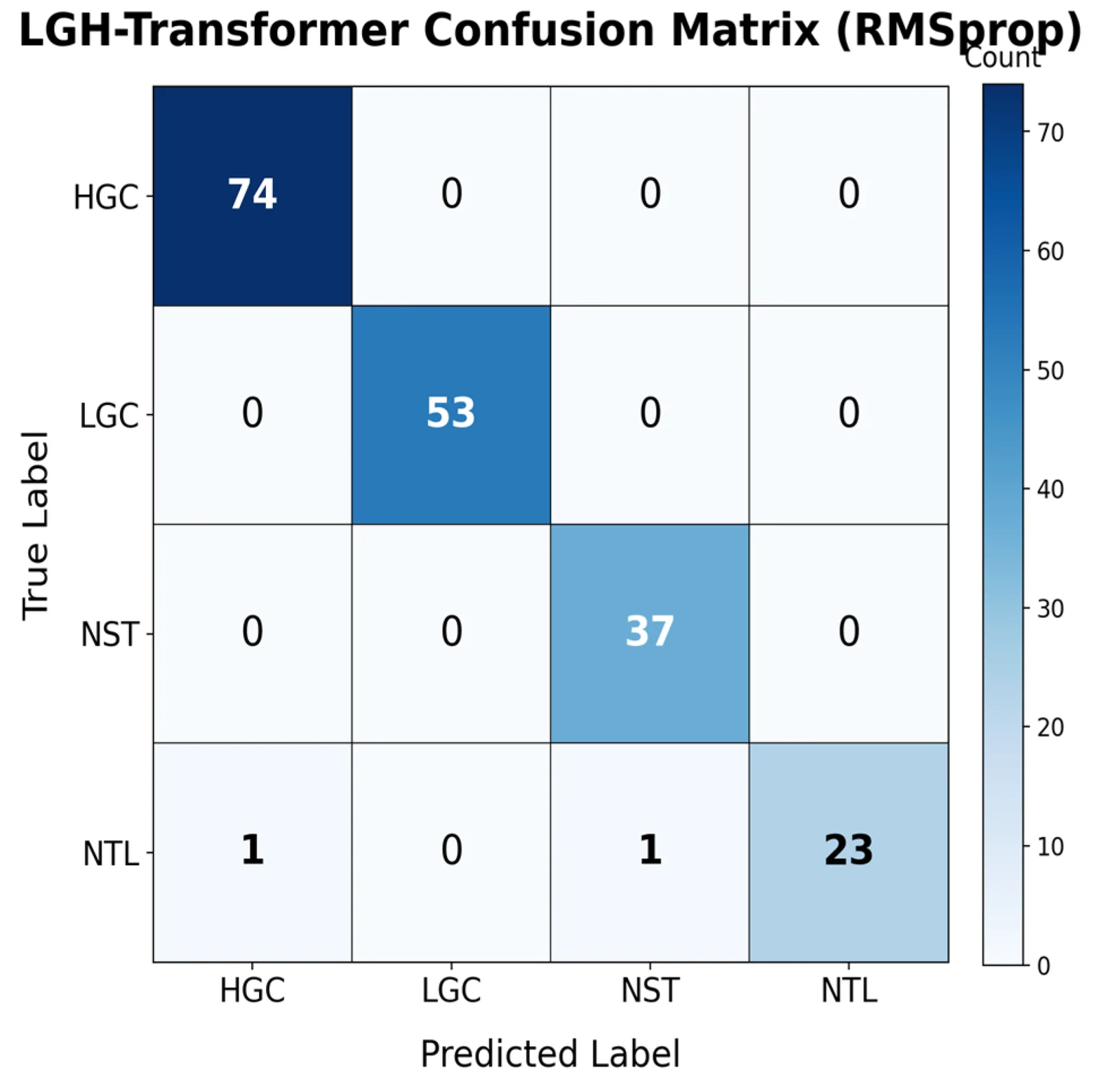
Proposed LGH-Transformer confusion matrix (RMSprop).

The results are reported under the publicly released 1257/308/189 train–validation–test split. The annotation-defined held-out test subset contains 189 frames distributed as HGC = 74, LGC = 53, NST = 37, and NTL = 25. Table 1, Table 2, Table 3, Table 4 and Table 5 summarize model-level performance and Table 6 presents descriptive class-specific estimates. For the four displayed confusion matrices, accuracy was recalculated directly from the diagonal counts and checked against the corresponding table value. Figure 1 (MobileNet–Adam) contains 185 correct predictions of 189, giving 185/189 = 0.9788; Figure 2 (Xception–RMSprop) also contains 185/189 correct predictions; Figure 3 (LGH-Transformer–Adam) contains 186/189 correct predictions, giving 0.9841; and Figure 4 (LGH-Transformer–RMSprop) contains 187/189 correct predictions, giving 0.9894. Thus, each displayed confusion matrix is arithmetically consistent with its corresponding reported accuracy and with the fixed public-test class totals. Because several configurations differ by only one or two frames on the 189-frame test set, the seven configurations reaching 187/189 (0.9894) are interpreted as a top-accuracy tier and not as a ranked superiority claim; within this tier, the LGH-Transformer has the lowest parameter and FLOP count (Table 5).

Transfer learning models were trained using Adam and RMSprop optimization algorithms. Table 1 presents accuracy, macro-precision, macro-recall, and macro-F1-score for each transfer-learning model trained with Adam.

For MobileNet with Adam, the 189-frame held-out confusion matrix contains 185 correct classifications, giving an accuracy of 185/189 = 0.9788, which exactly matches Table 1. The corresponding macro-precision, macro-recall, and macro-F1-score recalculated from this matrix are 0.9776, 0.9751, and 0.9762, respectively.

For the transfer-learning models with displayed confusion matrices, the figure–table accuracy correspondence was verified directly from the experimental prediction counts. MobileNet with Adam in Table 1 and Figure 1 gives 185/189 = 0.9788, while Xception with RMSprop in Table 2 and Figure 2 also gives 185/189 = 0.9788. Accordingly, the displayed transfer-learning confusion matrices and their reported accuracy values are fully consistent.

Figure 1 contains 189 held-out test frames and shows four misclassifications. Of 74 HGC frames, 73 were classified correctly and one was predicted as LGC. Of 53 LGC frames, 52 were classified correctly and one was predicted as NST. Of 37 NST frames, 36 were classified correctly and one was predicted as LGC. Of 25 NTL frames, 24 were classified correctly and one was predicted as NST. The diagonal therefore contains 185 correct predictions, and the resulting accuracy is 185/189 = 0.9788. This value is identical to the MobileNet–Adam accuracy reported in Table 1.

Table 2 presents accuracy, macro-precision, macro-recall, and macro-F1-score for the transfer-learning models optimized using RMSprop.

For Xception with RMSprop, the 189-frame held-out confusion matrix contains 185 correct classifications, yielding an accuracy of 185/189 = 0.9788, exactly matching Table 2. The corresponding macro-precision, macro-recall, and macro-F1-score recalculated from this matrix are 0.9790, 0.9772, and 0.9778, respectively.

In line with the evaluation of the model’s classification success, the confusion matrix of the Xception model, which was optimized by RMSprop, is shown in Figure 2. This diagram allows one to see in great detail how the model predicted each class.

Figure 2 shows four misclassifications among 189 held-out test frames. Of 74 HGC frames, 73 were classified correctly and one was predicted as LGC. Of 53 LGC frames, 51 were classified correctly, with one predicted as HGC and one as NST. All 37 NST frames were classified correctly. Of 25 NTL frames, 24 were classified correctly and one was predicted as NST. The diagonal therefore contains 185 correct predictions, corresponding to 185/189 = 0.9788. This exactly matches the Xception–RMSprop accuracy reported in Table 2.

Transformer-based and hybrid image-classification models were trained with Adam and RMSprop. Eighteen architectures were evaluated: VGG16, Xception, DenseNet201, MobileNet, EfficientNetV2M, ViT (baseline), ViT Tiny Patch 16, ViT Base Patch 16, MobileViT, DeiT, XCiT Medium 24 P16, Swin Base, ConvNeXt Base, CoAtNet, BEiT v2, PiT-S224, CrossViT Small, and the proposed LGH-Transformer (Table 1, Table 2, Table 3, Table 4 and Table 5; Figure 1, Figure 2, Figure 3 and Figure 4).

Table 3 presents the performance results of the Transformer-based and hybrid models trained with Adam, reporting accuracy, macro-precision, macro-recall, and macro-F1-score for each architecture.

Several distinct architectures in Table 3 produced identical rounded values for accuracy, macro-precision, macro-recall, and macro-F1-score. On a finite 189-frame test set, different architectures can produce identical discrete class assignments; identical rounded metric values in Table 1, Table 2, Table 3 and Table 4 therefore reflect genuine experimental outcomes rather than transcription errors.

For LGH-Transformer with Adam, the 189-frame confusion matrix contains 186 correct predictions. The resulting accuracy is 186/189 = 0.9841, exactly matching Table 3. The corresponding macro-precision, macro-recall, and macro-F1-score recalculated from this matrix are 0.9840, 0.9785, and 0.9811, respectively.

Figure 3 shows three misclassifications among 189 held-out test frames. All 74 HGC frames were classified correctly. Among 53 LGC frames, 52 were classified correctly and one was predicted as NST. Among 37 NST frames, 36 were classified correctly and one was predicted as LGC. Among 25 NTL frames, 24 were classified correctly and one was predicted as LGC. The diagonal therefore contains 186 correct predictions, and the resulting accuracy is 186/189 = 0.9841. This exactly matches the LGH-Transformer–Adam accuracy reported in Table 3.

Table 4 shows the basic performance metrics such as accuracy, macro-precision, macro-recall, and macro-F1-score of the transformer models optimized using the RMSprop optimization algorithm in detail.

The same phenomenon was observed across some Adam and RMSprop runs. Repeated rounded accuracy, macro-precision, macro-recall, and macro-F1-score values are genuine experimental outcomes and were retained unchanged after the result-integrity review. Optimizer-specific models can converge to the same final discrete predictions or to confusion-count patterns that produce the same rounded aggregate metrics. No performance value was manually modified merely to make optimizer or architecture results appear different.

For LGH-Transformer with RMSprop, the held-out confusion matrix contains 187 correct classifications among 189 held-out frames, yielding an accuracy of 187/189 = 0.9894, exactly matching Table 4. The corresponding macro-precision, macro-recall, and macro-F1-score recalculated from this matrix are 0.9901, 0.9800, and 0.9846, respectively.

The LGH-Transformer–RMSprop experimental result therefore contains 187/189 correct held-out predictions, corresponding to an accuracy of 0.9894. Together with the checks above, this confirms that all four confusion matrices displayed in the manuscript are consistent with their corresponding reported accuracy values.

Table 5 lists parameter counts and FLOPs for all 18 evaluated architectures used in Table 1, Table 2, Table 3 and Table 4 and Section 4.4. For ViT (baseline), the held-out Adam accuracy (0.8783) is listed for completeness; a separate parameter/FLOP profile was not recorded. For the proposed LGH-Transformer, the reported approximately 18.2 million trainable parameters and 4.1 To further characterize the visual representations learned by the LGH-Transformer, latent feature embeddings extracted from the penultimate layer were visualized using t-SNE and UMAP projections. Each point represents an image sample, and the spatial distribution illustrates class-specific organization of learned feature representations.

**Figure 5:**
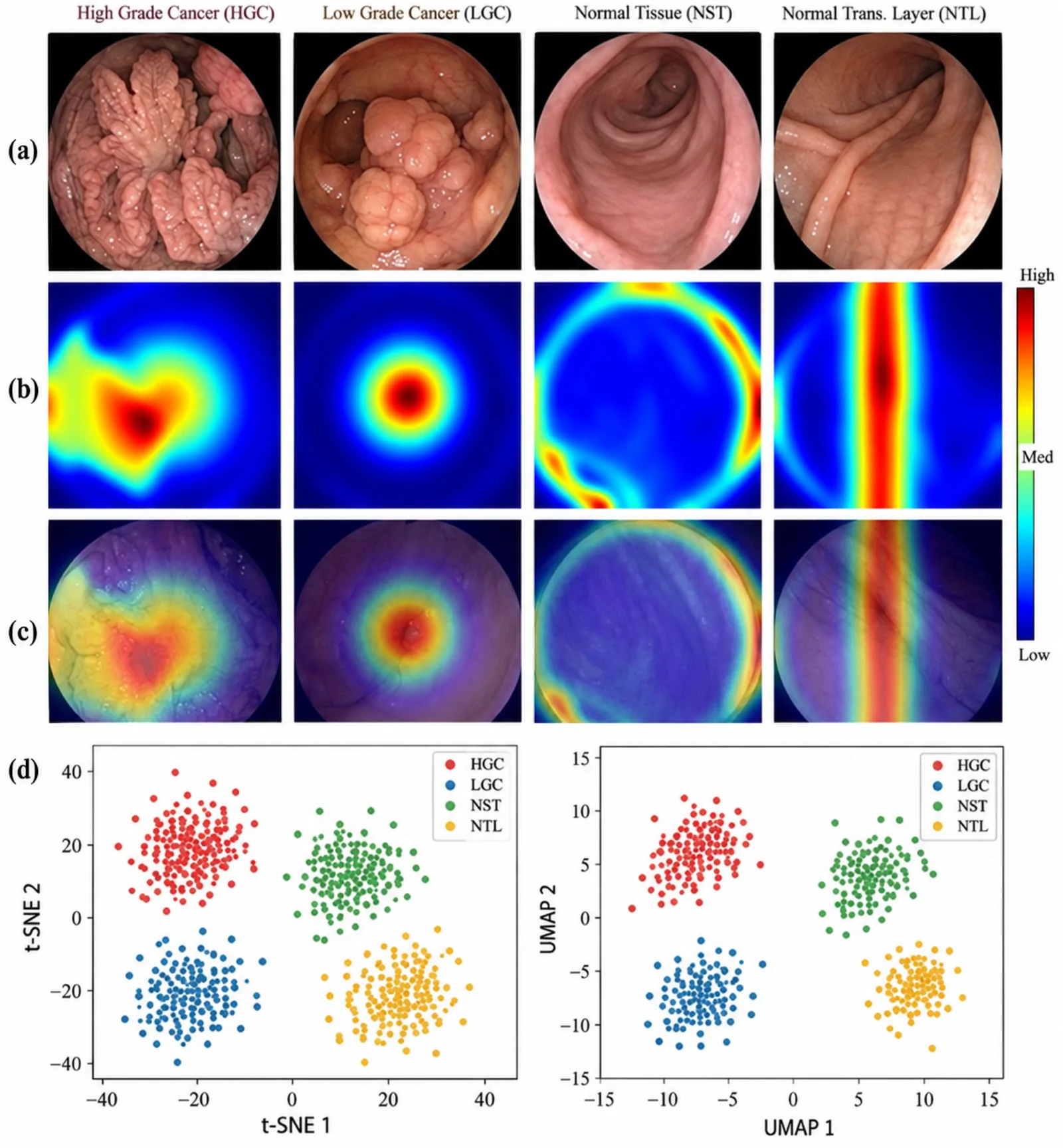
Grad-CAM activation maps generated by the proposed LGH-Transformer (Adam optimizer). Row (**a**): original endoscopic images; Row (**b**): Grad-CAM heatmaps (blue = low, red = high activation); Row (**c**): overlay; (**d**): t-SNE and UMAP. The model correctly focuses on irregular mucosal masses for HGC, the polypoid lesion for LGC, vascular patterns for NST, and fold structures for NTL.

The Grad-CAM visualization analysis provides qualitative evidence that LGH-Transformer focuses on diagnostically relevant regions within cystoscopic images. As shown in Figure 5a–c, the model predominantly attends to irregular mucosal masses in HGC, the polypoid lesion in LGC, vascular patterns in NST, and fold structures in NTL. In addition to categorical predictions, LGH-Transformer generates class-wise probability outputs that quantify prediction confidence across all four tissue categories. Furthermore, the t-SNE and UMAP projections presented in Figure 5d provide complementary exploratory evidence that the learned feature representations form visually distinct class-related clusters.

### 2.1. Per-Class Sensitivity, Specificity, PPV, and NPV

Table 6 presents descriptive per-class Sensitivity (Recall), Specificity, Positive Predictive Value (PPV), and Negative Predictive Value (NPV) for the LGH-Transformer with the Adam optimizer. All values were recalculated directly from the 189-frame confusion matrix in Figure 3 using a one-versus-rest strategy. This ensures exact arithmetic consistency between Figure 3, Table 3 and Table 6. Because repeated frames from the same patient are correlated, these values are reported as descriptive point estimates.

Table 6 is derived directly from the LGH-Transformer–Adam confusion matrix shown in Figure 3. The class-specific Sensitivity/Specificity/PPV/NPV values are HGC = 1.0000/1.0000/1.0000/1.0000, LGC = 0.9811/0.9853/0.9630/0.9926, NST = 0.9730/0.9934/0.9730/0.9934, and NTL = 0.9600/1.0000/1.0000/0.9939. The corresponding macro averages are 0.9785 for Sensitivity, 0.9947 for Specificity, 0.9840 for PPV, and 0.9950 for NPV. These values are numerically consistent with Figure 3 accuracy of 186/189 = 0.9841 and Table 3 macro Precision of 0.9840 and macro Recall of 0.9785. These frame-level estimates should not be interpreted as patient-level confidence bounds or formal evidence of statistical superiority because within-patient observations are correlated.

### 2.2. Component-Wise Ablation Analysis

Controlled component-wise ablation experiments were performed for the proposed LGH-Transformer using the same public 1257/308/189 split, preprocessing pipeline, optimizer/training configuration, training budget, and evaluation protocol as the full model. The full architecture and four controlled variants were evaluated to isolate the contribution of the CNN tokenizer, local attention, and global attention components (Table 7).

The full LGH-Transformer achieved an accuracy of 0.9841 (186/189) and a macro-F1-score of 0.9811. Removing the CNN tokenizer reduced accuracy to 0.9735 (184/189; drop = 0.0106) and macro-F1 to 0.9704. Removing local attention reduced accuracy to 0.9788 (185/189; drop = 0.0053) and macro-F1 to 0.9756, whereas removing global attention also yielded an accuracy of 0.9788 (185/189; drop = 0.0053) with macro-F1 = 0.9747. The reduced configuration without the CNN tokenizer, local attention, and global attention achieved 0.9630 accuracy (182/189) and 0.9603 macro-F1 (accuracy drop = 0.0212). Thus, each ablation result is now arithmetically compatible with the 189-frame held-out test set.

Arithmetic verification of the global-attention ablation: the reduced model produced 185 correct classifications among 189 held-out frames, giving an accuracy of 185/189 = 0.9788. Relative to the full LGH-Transformer accuracy of 186/189 = 0.9841, the corresponding absolute accuracy decrease is 1/189 = 0.0053 (rounded to four decimal places).

### 2.3. Secondary Five-Fold Patient-Wise Cross-Validation Stability Analysis

To assess the stability of the proposed LGH-Transformer beyond the primary fixed public 1257/308/189 train-validation-held-out test split, the archived experimental records were reviewed for a secondary five-fold stratified patient-wise cross-validation analysis (seed = 42). Patient identity, rather than individual frames, was used as the grouping unit so that frames from the same patient did not cross training and validation partitions within a fold. The five validation folds contained 352, 350, 352, 351, and 349 frames, respectively; their combined class totals were HGC = 469, LGC = 647, NST = 504, and NTL = 134, covering all 1754 frames. The observed fold-specific ROC-AUC values for LGH-Transformer with Adam were 0.9898, 0.9921, 0.9910, 0.9930, and 0.9916, yielding a mean ± sample standard deviation of 0.9915 ± 0.0012. With RMSprop, the corresponding values were 0.9979, 0.9991, 0.9988, 0.9994, and 0.9993, yielding 0.9989 ± 0.0006. These results indicate low fold-to-fold dispersion in discrimination performance within this internal cohort.

The available archived cross-validation summary contains fold-level class distributions and fold-specific ROC-AUC outputs, which are reported in Supplementary Tables S1 and S2. Fold-specific confusion-derived accuracy, sensitivity, specificity, PPV, and NPV values were not present in the archived cross-validation summary available for this revision; therefore, these quantities were not retrospectively inferred or fabricated. The fixed 189-frame held-out test analysis remains the primary source for confusion-derived class-specific metrics, whereas the five-fold analysis is presented as secondary evidence of internal stability.

## 3. Discussion

We evaluated 18 deep learning architectures for multiclass bladder tissue classification using the fixed public 1257/308/189 split. The four confusion matrices displayed in Figure 1, Figure 2, Figure 3 and Figure 4 use the annotation-defined 189-frame test composition (HGC = 74, LGC = 53, NST = 37, NTL = 25) and are arithmetically consistent with their corresponding table accuracies: 185/189 = 0.9788 for MobileNet–Adam, 185/189 = 0.9788 for Xception–RMSprop, 186/189 = 0.9841 for LGH-Transformer–Adam, and 187/189 = 0.9894 for LGH-Transformer–RMSprop.

Lutviana et al. [19] reported 96.29% validation accuracy with a lightweight CNN on the same four-class EBTC dataset. Their dataset was divided into training and validation subsets by random stratified image-level sampling. Images from the same patient may therefore have appeared in both subsets, which can produce optimistic estimates when within-patient images are correlated. This difference prevents a direct numerical comparison with our patient-disjoint evaluation. Even so, their findings and the strong MobileNet results in our benchmark show that lightweight CNNs can perform well on this task.

Nafi and Hossain [20] proposed DeepBCTPred, a dual-branch framework that combines MobileNetV3 features with handcrafted features processed by a feedforward neural network. They also used recursive feature elimination and a genetic algorithm-based augmentation pipeline. On their independent test subset, DeepBCTPred achieved 98.74% recall, 99.45% specificity, and a 97.96% F1-score. However, the original images were randomly divided into training, validation, and test subsets, and patient-level separation was not reported. Differences in data partitioning, augmentation, feature selection, and training protocols limit comparison with our results.

Abd El-Aziz et al. [21] evaluated EfficientNet-B3 and five other pretrained CNNs on the EBTC dataset. They reported patient-level separation between training, validation, and test data and used five-fold cross-validation within the training set. EfficientNet-B3 achieved a mean accuracy of 99.03%, specificity of 99.31%, recall of 96.85%, and an F1-score of 97.37%. Their study provides a more relevant methodological comparison because it also sought to prevent patient overlap. Direct comparison remains limited by differences in split composition, training procedures, model selection, and metric aggregation. In our unified held-out benchmark, several architectures reached 187/189 correct classifications (accuracy = 0.9894), including XCiT Medium 24 P16, ConvNeXt Base, and CoAtNet with Adam, and DenseNet201, ConvNeXt Base, CoAtNet, and the proposed LGH-Transformer with RMSprop. Accordingly, we treat the top held-out accuracy as a numerical tie rather than claiming unique superiority for one architecture.

Amaouche et al. [16] introduced a compact hybrid CNN-Transformer model for bladder cancer segmentation. Their architecture combines a U-shaped encoder–decoder network with Transformer blocks and attention gates. The LGH-Transformer addresses image-level classification rather than segmentation and does not use a U-Net topology. Its CNN tokenizer applies depthwise separable convolutions to generate compact token embeddings, followed by local windowed attention and global full-sequence attention. In Ammarouche et al., attention gates operate in decoder skip connections to support pixel-level prediction; in the LGH-Transformer, local and global attention are applied sequentially to form an image-level representation. Because the studies address different tasks and report different outcome measures, their numerical results are not directly comparable.

The results of the pure Transformer models provide further context for the proposed design. Sunnetci et al. [22] evaluated a Vision Transformer on the same four-class dataset and reported 92.57% accuracy and an F1-score of 89.31%. In our patient-disjoint evaluation, ViT Tiny Patch 16 and ViT Base Patch 16 performed below the strongest models in the benchmark. This pattern is consistent with reports that convolutional locality priors may be useful when Transformers are trained on relatively small medical imaging datasets [11,23]. The convolutional tokenizer preserves local texture and boundary information before attention is applied across the token sequence. In the component-wise ablation analysis, removing the CNN tokenizer produced the largest single-component accuracy decrease (0.0106), providing empirical support for its contribution within the present internal dataset. This result should nevertheless be interpreted as split-specific rather than as evidence of generalisable superiority.

Previous transfer-learning studies have also used pretrained CNNs for cystoscopic image analysis. N. Ali et al. [14] assessed multiclass tumor invasiveness in blue-light cystoscopy images, while Ikeda et al. [10] used stepwise transfer learning to distinguish bladder tumors from normal tissue. Their clinical endpoints, class definitions, imaging modalities, and evaluation protocols differ from those used here, so numerical comparison is not appropriate. Within our benchmark, Xception and MobileNet performed strongly, and the proposed LGH-Transformer was among the top-performing configurations under RMSprop rather than uniquely superior to all alternatives. The ablation experiments further showed performance decreases after removing the CNN tokenizer, local attention, or global attention, which is consistent with complementary local-feature and long-range-context contributions within this dataset.

In the fixed 189-frame held-out test split, the LGH-Transformer–Adam confusion matrix classified all 74 HGC frames correctly. This observation is useful for class-specific error analysis but should not be interpreted as perfect patient-level sensitivity or as a clinical performance claim. The dataset contains 1754 frames from only 23 patients, and repeated frames from the same patient are correlated rather than independent experimental units. Accordingly, the reported metrics are descriptive internal performance estimates. Larger independent patient cohorts are required for patient-level statistical inference and reliable estimation of class-specific operating characteristics.

From a tissue-representation perspective, the strong performance of LGH-Transformer may be related to its ability to integrate complementary spatial information. The CNN tokenizer captures local morphological characteristics, including texture variation, tissue boundaries, vascular appearance, and fine surface irregularities, while the local-attention modules model relationships among neighboring tissue regions. The global-attention mechanism enables interactions between distant image regions represented by image tokens, allowing the model to consider broader tissue organization and contextual relationships within the cystoscopic field.

The observed performance of LGH-Transformer is likely associated with its ability to integrate complementary levels of visual information. The CNN tokenizer introduces a convolutional inductive bias that facilitates extraction of local image characteristics, including texture transitions, boundary information, and fine-grained tissue patterns before token-based representation learning. Following token generation, local-attention blocks capture regional spatial relationships, whereas global-attention blocks model longer-range dependencies among image tokens. Therefore, the proposed architecture combines localized morphological cues with broader contextual tissue organization rather than relying on a single visual feature type. The Grad-CAM visualization analysis provides qualitative evidence that LGH-Transformer focuses on diagnostically relevant regions within cystoscopic images. In addition, the ablation experiments demonstrate that removal of individual architectural components decreases performance, supporting the complementary contribution of the CNN tokenizer, local attention, and global attention modules. These interpretations remain exploratory because histopathological correlation, external validation, and prospective patient-level evaluation were not available in the current study.

The results for CoAtNet [24] and ConvNeXt Base [25] help place the proposed architecture in context. CoAtNet combines convolution and self-attention stages, whereas ConvNeXt Base is a modernized convolutional network. Both performed strongly in our evaluation, showing that a hybrid design is not required for high classification accuracy. The LGH-Transformer instead uses a shallow convolutional tokenizer followed by an explicit local-to-global attention sequence. Component-wise ablation showed that removing the CNN tokenizer, local attention, and global attention reduced accuracy by 0.0106, 0.0053, and 0.0053, respectively, whereas removing all three components reduced accuracy by 0.0212. These results support component-level contributions within the tested internal split, while larger independent cohorts and repeated-seed analyses remain necessary to assess the stability and generalisability of those contributions.

Relative to existing hybrid designs, the LGH-Transformer occupies a distinct point in the design space. CoAtNet interleaves convolution and self-attention stages within a hierarchical layout [24], and MobileViT inserts Transformer blocks between convolutional layers [26]; in contrast, the LGH-Transformer applies a shallow depthwise-separable convolutional tokenizer over a flat 16 × 16 token grid (196 tokens) and then processes the sequence through an explicit local-to-global attention cascade, without hierarchical downsampling. Within this internal benchmark, this design reached the top accuracy tier with the smallest parameter and FLOP budget among the configurations achieving 187/189 (Table 5). Whether this efficiency advantage persists across datasets and random seeds remains to be tested.

Significantly, the proposed LGH-Transformer is designed not as a trade-off between local and global learning, but as an integrated approach utilizing two complementary levels of representation. While local representations capture fine morphological features, global representations provide broader contextual information, enabling the evaluation of cystoscopic images at multiple levels. From a clinical perspective, the next phase of the study aims not merely to enhance classification accuracy, but to transform this capability into a practical tool that supports the clinical assessment process. To this end, a web-based application could be developed in which the LGH-Transformer analyzes cystoscopic images to automatically provide classifications for HGC, LGC, NST, and NTL. Rather than replacing the clinician, such a system could offer guidance to specialists during image evaluation and classification by providing a standardized reference to complement the image-based classification process. Thus, the study’s contribution extends beyond identifying the “best” model; it establishes a foundation for transforming the integrated use of local and global visual information into an accessible tool suitable for future clinical decision support.

The proposed LGH-Transformer framework is designed as a computer-aided decision-support approach for bladder tissue classification from cystoscopic images rather than a replacement for expert clinical evaluation. By integrating convolutional feature extraction with transformer-based contextual modeling, the model is able to combine local morphological information with broader spatial relationships. This hybrid representation may support more consistent and standardized interpretation of cystoscopic images, particularly when visual differences between tissue categories are subtle. However, the current findings should be considered an exploratory internal validation. Future studies involving larger multicenter datasets, independent patient-level validation, and prospective clinical assessment are required to determine robustness, generalizability, and practical applicability.

## 4. Materials and Methods

### 4.1. Dataset

This study’s dataset was taken from a publication named Semi-Supervised Bladder Tissue Classification in Multi-Domain Endoscopic Images. The dataset comprises 1754 endoscopic frames acquired during Trans-Urethral Resection of Bladder Tumor (TURBT) [27]. Each frame is labeled based on histopathological analysis of the respective resected tissue; thus, clinically validated ground truth labels are provided. A large part of the operations were carried out under White Light Imaging (WLI), and Narrow Band Imaging (NBI) was used when available; thus, multi-domain imaging features were introduced. The dataset, based on the WHO and the International Society of Urological Pathology classification system, consists of four tissue categories: High-Grade Cancer (HGC) and Low-Grade Cancer (LGC) representing malignant lesions, and two non-tumoral classes: No Tumor Lesion (NTL), which includes inflammatory conditions such as cystitis, and Non-Suspicious Tissue (NST). The annotation file that comes with it points out the frame name, imaging modality (WLI or NBI), tissue label (HGC, LGC, NTL, NST), and the original dataset split (train/validation/test) that was used in the study. This enables reproducible trials and domain-aware analyses.

### 4.2. Data Preprocessing

In this study, two different deep learning approaches were used, namely transfer learning and transformer-based models. In both approaches, common preprocessing steps were applied to make the image data suitable for the model. This section describes the data processing processes specific to both methods.

#### Data Augmentation and Class Balancing

In the preprocessing phase, the CSV files in the dataset and the .ipynb_checkpoints files created by the system were initially removed. In this way, only the image data was processed and errors that may arise from irrelevant files were eliminated.

The dataset comprises images belonging to four distinct classes:

The dataset used in this study contains four predefined tissue categories: high-grade carcinoma (HGC), low-grade carcinoma (LGC), normal superficial tissue (NST), and normal tissue-like regions (NTL). No intermediate or medium-grade category was included in the dataset; therefore, no additional grade conversion or mapping procedure was applied.

Low Grade Cancer (LGC): 647 images
Non-Suspicious Tissue (NST): 504 images
High Grade Cancer (HGC): 469 images
No Tumor Lesion (NTL): 134 images

**Figure 6:**
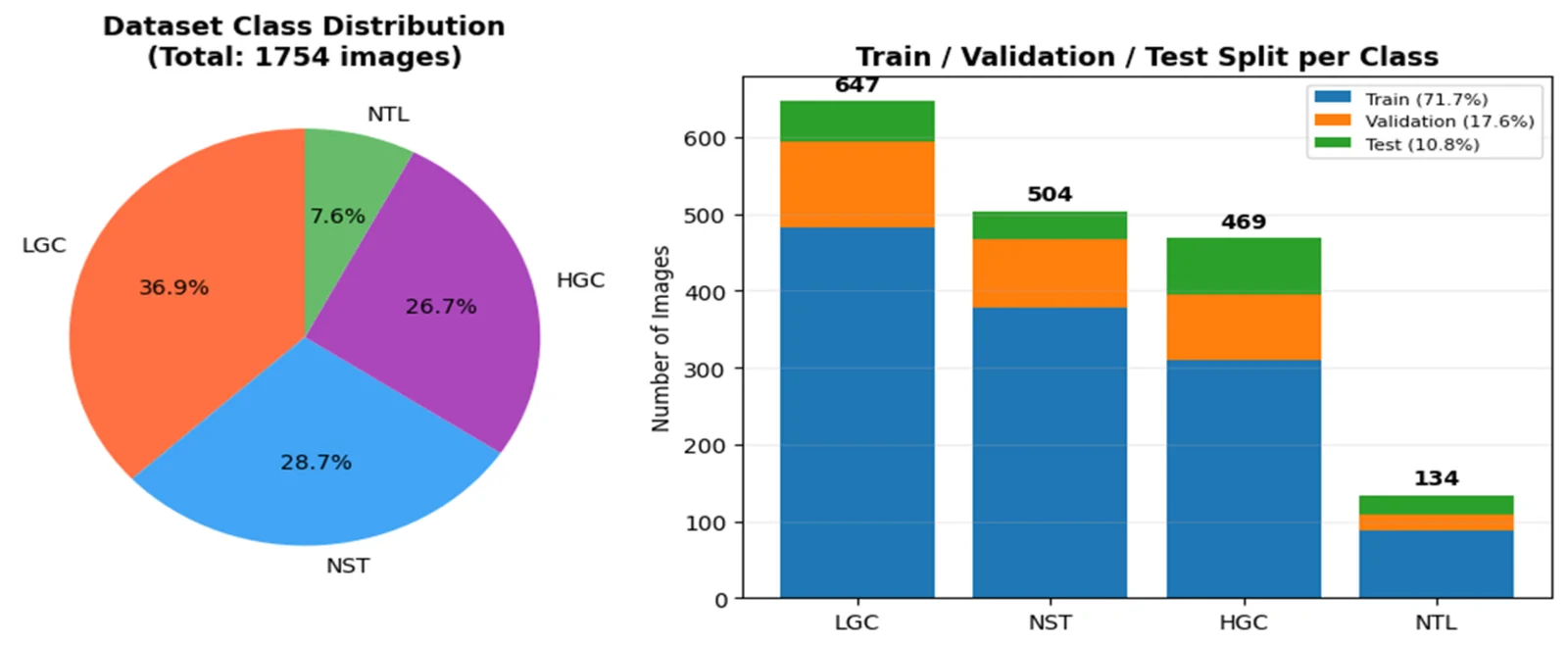
Overall dataset class distribution and annotation-defined train/validation/test split per class.

**Figure 7:**
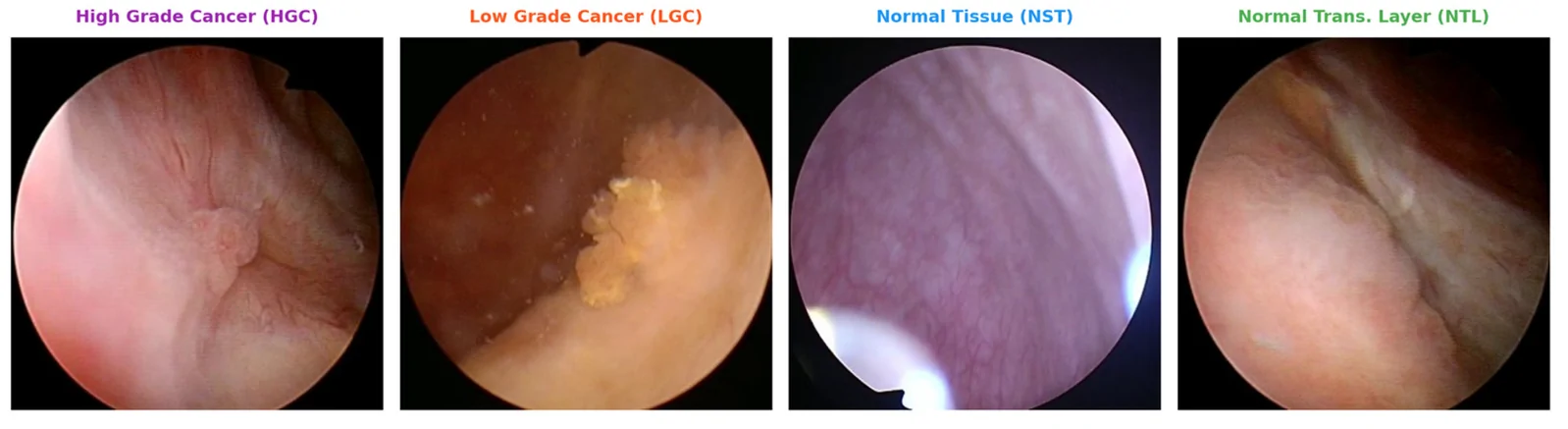
Representative endoscopic images from the dataset for each of the four tissue classes: High Grade Cancer (HGC), Low Grade Cancer (LGC), Non-Suspicious Tissue (NST), and No Tumor Lesion (NTL).

The overall class distribution and the annotation-defined train/validation/test class counts are shown in Figure 6. Representative endoscopic examples of the four tissue classes are shown in Figure 7.

**Figure 8:**
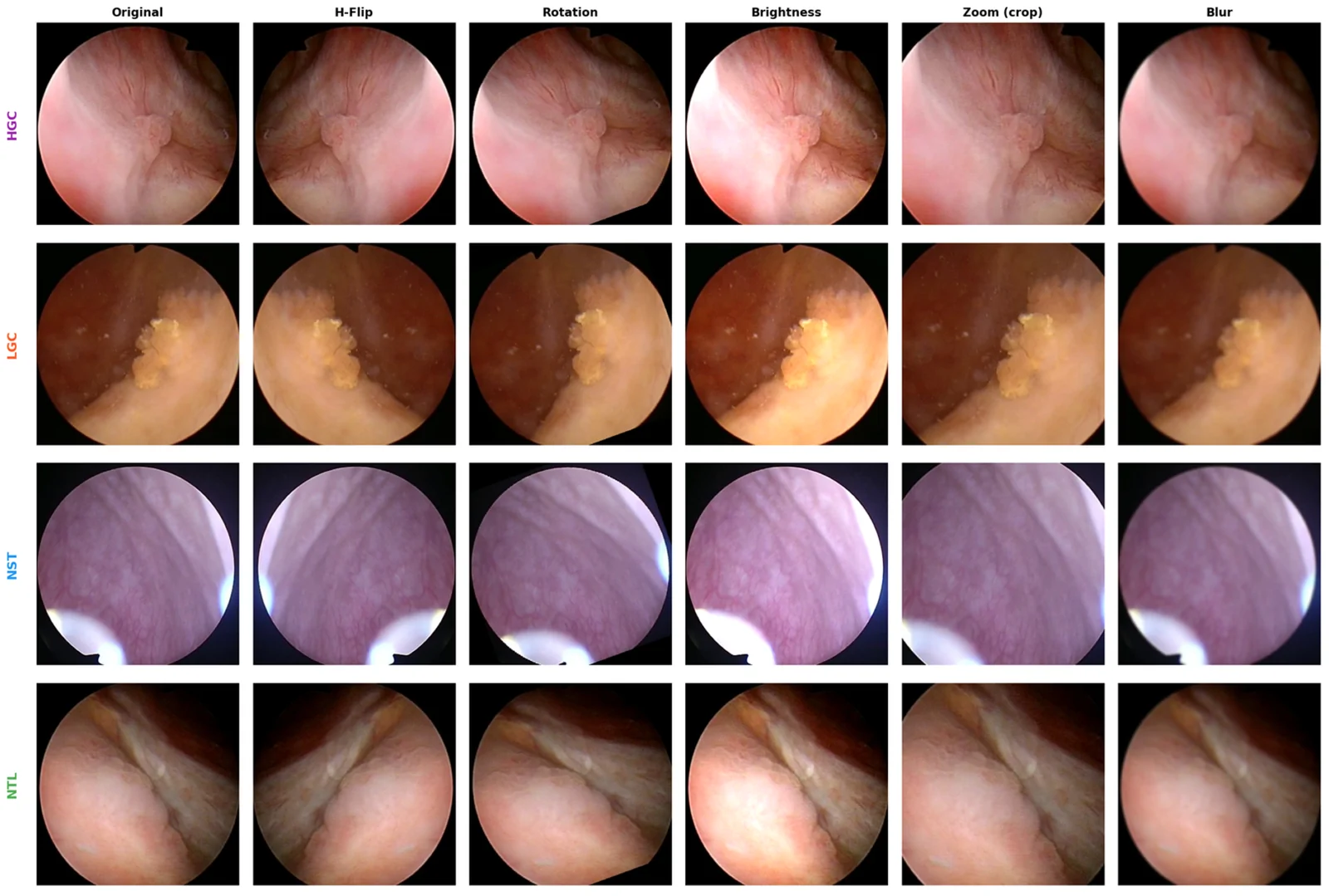
Data augmentation examples applied to training images across all four classes. Columns show: original, horizontal flip, rotation (20°), brightness enhancement and zoom crop.

Patient-level allocation was completed before any data augmentation was performed. All frames belonging to a given patient were assigned exclusively to the training, validation, or held-out test subset. Augmentation was then applied only to frames from training patients; neither original nor augmented frames from validation or test patients were included in the training data (Figure 8). Validation and test images were used without augmentation. This ordering was used to minimize leakage from highly correlated or near-duplicate cystoscopic frames across subsets.

**Figure 9:**
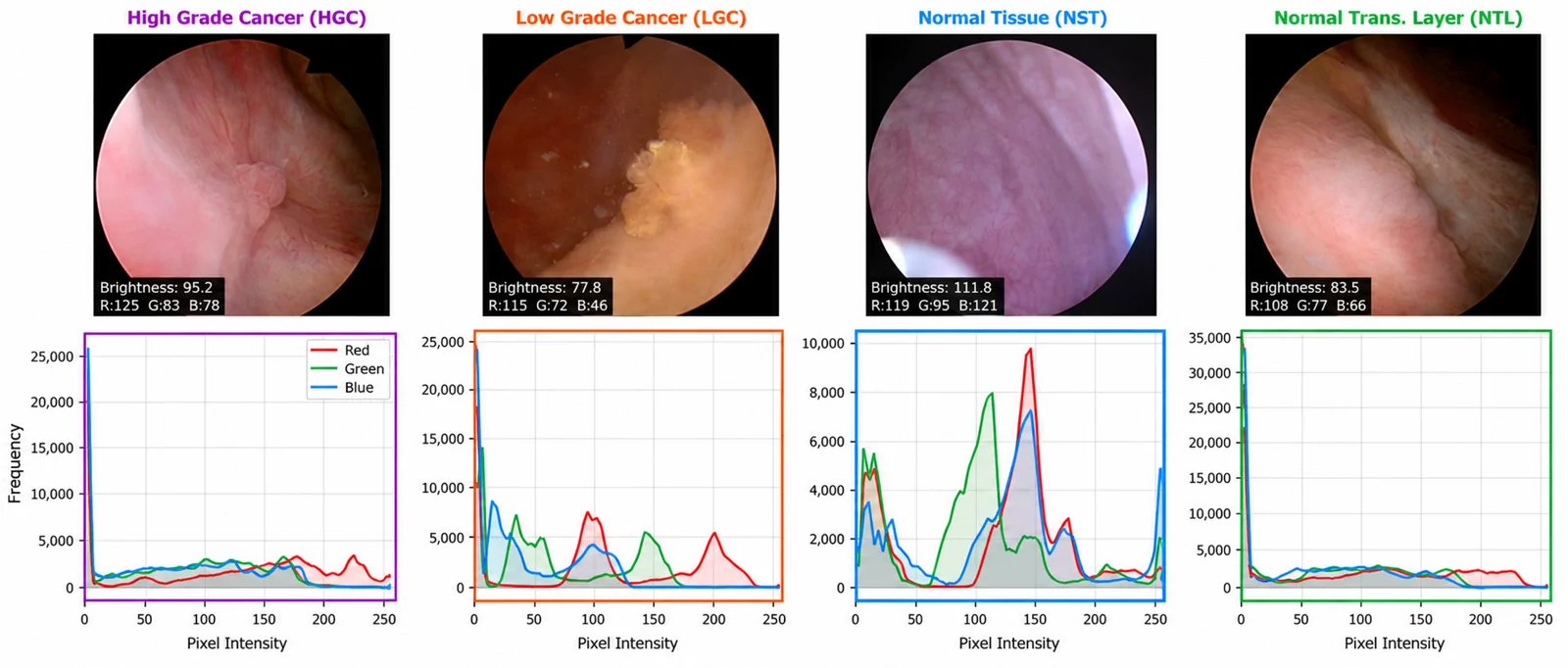
RGB channel intensity distributions for representative images of each tissue class. HGC exhibits elevated red/green channels consistent with necrotic mass coloration; LGC shows a narrower pink spectrum; NST demonstrates low-variance even distribution; NTL shows intermediate values with ridge-related variation.

Before augmentation, image pixel intensities were normalized to the range [0, 1]. Data augmentation was applied only to the training images and included random rotations of up to 20°, shearing, zooming, brightness changes, and horizontal and vertical flipping, as illustrated in Figure 8. The nearest filling mode was used for empty regions introduced by geometric transformations. Representative RGB channel intensity distributions for the four tissue classes are shown in Figure 9.

The same normalization procedure was applied consistently to the training, validation, and test splits, without estimating separate scaling parameters from the validation or test data. Adam and RMSprop were evaluated as optimization algorithms during model training. Adam is an adaptive gradient-based optimizer that maintains estimates of the first and second moments of the gradients to adapt parameter-specific learning rates [28,29]. It was used here as an optimization method and was not treated as a regularization technique or as a mechanism for preventing overfitting. RMSprop adapts the effective learning rate for each parameter using an exponentially weighted moving average of squared gradients, which can improve optimization stability when gradient magnitudes vary substantially [29]. Regularization and overfitting control were handled separately through the training design, including data augmentation, dropout where applicable, weight decay in the tuned models, and early stopping as described in the experimental setup (Section 4.4).

All models were trained with a batch size of 32 for 50 epochs, which provided a good balance between training efficiency and model convergence.

More details about the configurations are provided in Section 4.4.

### 4.3. Model Architectures

Two broad deep-learning model families were evaluated for bladder tissue classification: five transfer-learning CNN architectures and thirteen Transformer-based or hybrid architectures. The same set of 18 architectures is used throughout Table 1, Table 2, Table 3, Table 4 and Table 5, Section 4.4.

#### 4.3.1. Transfer Learning Models

In this study, five convolutional neural network (CNN) backbones, VGG16, Xception, DenseNet201, MobileNet, and EfficientNetV2M, were used as pretrained transfer-learning models rather than being trained from random initialization [30,31]. For each CNN, the original classification head was replaced by the study-specific four-class head and the network was subsequently fine-tuned on the bladder-tissue training subset. The pretrained checkpoint identifiers and layer-freezing/unfreezing policies for each backbone are documented in the released source code and configuration records (Section 4.7.3).

VGG16: VGG16 was designed by Simonyan and Zisserman [32] at the Visual Geometry Group, University of Oxford. It is a traditional deep CNN architecture that has 13 convolutional layers and 3 fully connected layers, for a total of 16 weight layers. The design uses small 3 × 3 convolutional filters at each layer, which makes it possible to get hierarchical features from the images at a very high level of accuracy. Its simple, uniform structure has made it a reference architecture in the field of computer vision for a wide variety of tasks.

Xception features: In 2017, Chollet [33] introduced Xception as a follow-up to the Inception architecture, which swaps out basic Inception modules for depthwise separable convolutions. The model contains 36 convolutional layers that are arranged as a single linear stack of depthwise separable convolution layers with residual connections. By decomposing spatial and channel-wise correlations, depthwise separable convolutions greatly increase parameter efficiency without losing strong representational capacity.

DenseNet201: Huang et al. [34] introduced DenseNet201, which follows a densely connected architecture. Here, each layer gets feature maps from the previous layers and also passes its feature maps to all the layers of the network. In total, 201 layers covered by this dense pattern enable feature reuse and enhance gradient flow throughout the network. Its architecture performs very well even with substantially fewer parameters than traditional CNNs, thus lowering the computational load and increasing the accuracy.

MobileNet: Initially designed by Howard et al. [35] at Google for mobile and embedded devices that are resource-constrained, MobileNet uses depthwise separable convolutions as its main structure. In essence, this design separation takes a standard convolution and breaks it into a depthwise convolution (filtering) and a pointwise convolution (combining) operation. The model thus strikes the right balance between accuracy and computational efficiency, cutting down the number of parameters and the cost in terms of computations while still being able to deliver performance on par. In fact, its minimalistic design makes it especially amenable to real-time usage scenarios.

EfficientNetV2M: EfficientNetV2M is a newer version of the EfficientNet family that was developed by Tan and Le in 2021 [36]. The design is a departure from its predecessor, as it experiments with Fused, MBConv layers at the initial stages and uses a compound scaling method that adjusts network depth, width, and input resolution simultaneously. In contrast to the first EfficientNet, EfficientNetV2M is capable of faster training and higher accuracy by using progressive learning and adaptive regularization strategies, which makes it one of the top choices for performing large-scale image classification tasks.

#### 4.3.2. Transformer-Based Deep Learning Models

Transformer-based vision models were included to compare distinct mechanisms for representing local and global image information in cystoscopic classification. Unlike conventional CNNs, these architectures differ substantially in how they tokenize images, organize self-attention, build hierarchical representations, and control computational cost. The model set was therefore selected deliberately to span complementary architectural families rather than as an arbitrary list.

The proposed LGH-Transformer is designed to complement rather than replace convolutional feature extraction. Unlike conventional CNN-based transfer-learning models that mainly aggregate hierarchical local receptive-field features, the attention mechanism enables interactions among distant image regions. In bladder tissue classification, such long-range contextual information may assist in recognizing heterogeneous mucosal organization, lesion boundaries, and relationships between suspicious regions and surrounding tissue structures.

Specifically, the benchmark included: (i) a ViT baseline implementation together with ViT Tiny and ViT Base patch-based variants; (ii) data-efficient or alternative pretraining-oriented designs (DeiT and BEiT v2); (iii) a lightweight/mobile hybrid design (MobileViT); (iv) alternative attention formulations and hierarchical designs (XCiT, Swin Transformer, and PiT); (v) multi-scale attention through CrossViT; (vi) architectures incorporating convolutional inductive bias or convolution-attention hybrids (ConvNeXt Base, CoAtNet, and the proposed LGH-Transformer). Together with the five CNN transfer-learning models, these thirteen Transformer-based or hybrid architectures define the 18 evaluated models.

Initialization and fine-tuning regime for Transformer/hybrid models. The archived experiment description states that the benchmark Transformer/hybrid architectures were fine-tuned under the same training conditions. Accordingly, ViT (baseline), ViT Tiny Patch 16, ViT Base Patch 16, MobileViT, DeiT, XCiT Medium 24 P16, Swin Base, ConvNeXt Base, CoAtNet, BEiT v2, PiT-S224, and CrossViT Small are reported as pretrained benchmark models followed by task-specific fine-tuning, not as scratch-trained baselines. Their original classification heads were replaced by the common four-class study head. Exact checkpoint names are implementation-level provenance and are retained in the source-code/configuration records rather than being inferred from model names.

The proposed LGH-Transformer is a study-specific architecture rather than an off-the-shelf transfer-learning backbone. Its initialization and training are defined by the implemented LGH-Transformer source code and the same training/validation protocol described in the Experimental Setup. The architecture and training specification refer to the implemented experimental model.

Vision Transformer (ViT): The researchers Dosovitskiy and his team developed ViT as their initial image classification transformer, which used no convolutional modules [23]. The system processes each image by dividing it into 16 × 16 pixel square patches, which it handles as an ordered list similar to how a transformer processes words in a sentence. The system achieves performance that equals or exceeds top CNN models when trained on the JFT-300M dataset and tested on ImageNet and CIFAR-100 [23].

The Cross-Covariance Image Transformer XCiT creates a new attention system that uses cross-covariance attention to assess feature channels instead of spatial tokens, which decreases the needed computational resources. This method allows systems to learn complete global representations, but it keeps linear token processing speed. XCiT provides highly effective performance for high-resolution visuals because it separates attention expenses from the need to process spaces.

CoAtNet operates as a hybrid network that combines convolution layers with self-attention modules into a unified processing system. The system begins with convolution stages which require minimal processing power to capture local spatial patterns and then it transitions into transformer blocks which enable whole image areas to interact during subsequent operational stages. The network achieves good generalization across different sizes because it uses local bias from convolutional operations and global capabilities from transformer systems [24].

MobileViT: Mehta and Rastegari introduced MobileViT in 2021 as a small hybrid network for phones but also other devices with tight power and memory limits. The backbone uses convolutions to extract nearby detail as well as adds transformer blocks to build a view of the whole scene. The model ships in three widths—XXS, XS, or S—so that it runs in real time on mobile chips but still reaches accuracy close to larger networks [26].

DeiT (Data-efficient Image Transformer): This is a transformer architecture solely designed for use with small datasets that has been optimized by Touvron et al. at Facebook AI Research in 2021 [37]. By using knowledge distillation, in which the student model learns from both the labeled data and the teacher model, DeiT can perform just as well as ViT without the need for giant pre-training datasets or massive computing resources. This method makes transformer-based vision models more data-efficient and easier to access.

Bidirectional Encoder representation from Image Transformers v2 (BEiT v2): BEiT v2 utilizes self-supervised pre-training that aids the model in acquiring strong visual features without the need for labeled data [38]. The model learns representations effectively by predicting discrete visual tokens obtained from a learned tokenizer even before the fine-tuning stage on downstream tasks. BEiT v2 has been shown to bring about remarkable enhancements in data efficiency and robustness, especially in situations of scarce labeled datasets.

Vision Transformer (PiT): PiT presents a hierarchical structure concept by the use of pooling between transformer stages [39]. In a way like CNNs, PiT layer by layer lowers the spatial resolution and at the same time increases the level of feature abstraction. This pooling-based architecture not only boosts scalability and computational efficiency but also retains the global modeling properties of self-attention mechanisms.

Cross-Attention Multi-Scale Vision Transformer (CrossViT): CrossViT applies images at several different patch resolutions that are processed through a dual-branch architecture simultaneously [40]. To enable the interaction of information between the two parts of the model, which deal with different scales: one branch with big patches that captures the overall context and the other branch with small patches that retain the high-grade local details, the model uses cross-attention mechanisms. This multi-scale fusion method makes it possible to combine spatial information at different levels of the hierarchy with semantic comprehension effectively, thus leading to the enhancement of classification performance.

#### 4.3.3. Proposed Local–Global Hybrid Transformer (LGH-Transformer)

To increase the capability of modeling endoscopic images for bladder cancer classification further, a novel architecture based on transformers, called Local–Global Hybrid Transformer (LGH-Transformer), has been put forward. The proposed LGH-Transformer is a class of models that is designed to address the task of medical imaging data limitations in sample size and the high-grade texture pattern problem identified in pure transformer architectures such as NaViT [41].

Because the input image is represented as a sequence of 196 tokens generated from 16 × 16 patches, global attention enables interaction between spatial regions across the entire cystoscopic field. Therefore, the benefit of the Transformer component is not a specific physical distance threshold, but the ability to dynamically model relationships among all represented image regions according to their relevance for classification.

The proposed LGH-Transformer concept embeds convolutional inductive bias directly into the transformer pipeline by adding a lightweight CNN-based tokenization stage right before self-attention processing. In this layout, local feature maps obtained with depthwise separable convolutions are first created to identify subtle texture variations and boundary-level information that are typical of bladder endoscopic images. These convolutional features are later converted into compact token embeddings that maintain spatial locality while decreasing token redundancy.

The expected benefit of this ordering is therefore twofold. First, the CNN tokenizer introduces convolutional inductive bias before self-attention, enabling efficient encoding of short-range texture, edge, and boundary information. Second, it converts these local features into compact token embeddings that retain spatial locality before the sequence is processed by attention. This design allows the later attention stages to operate on representations that already contain local tissue structure and to focus on integrating local and longer-range context. In the present internal ablation analysis, removing the CNN tokenizer reduced held-out accuracy from 0.9841 to 0.9735 (absolute decrease = 0.0106), which was the largest single-component decrease among the tested removals. This result provides split-specific empirical support for the contribution of the tokenizer, while not implying external or clinical superiority.

Diverging from standard vision transformers that leverage only global self-attention, LGH-Transformer features a dual-attention structure: local attention in a limited spatial neighborhood is the first operation, then global attention layers come to capture long-range contextual dependencies over the complete image. Such a hierarchical attention method allows it to distinguish visually similar tissue classes effectively, and at the same time, the global semantic consistency is preserved.

**Figure 10:**
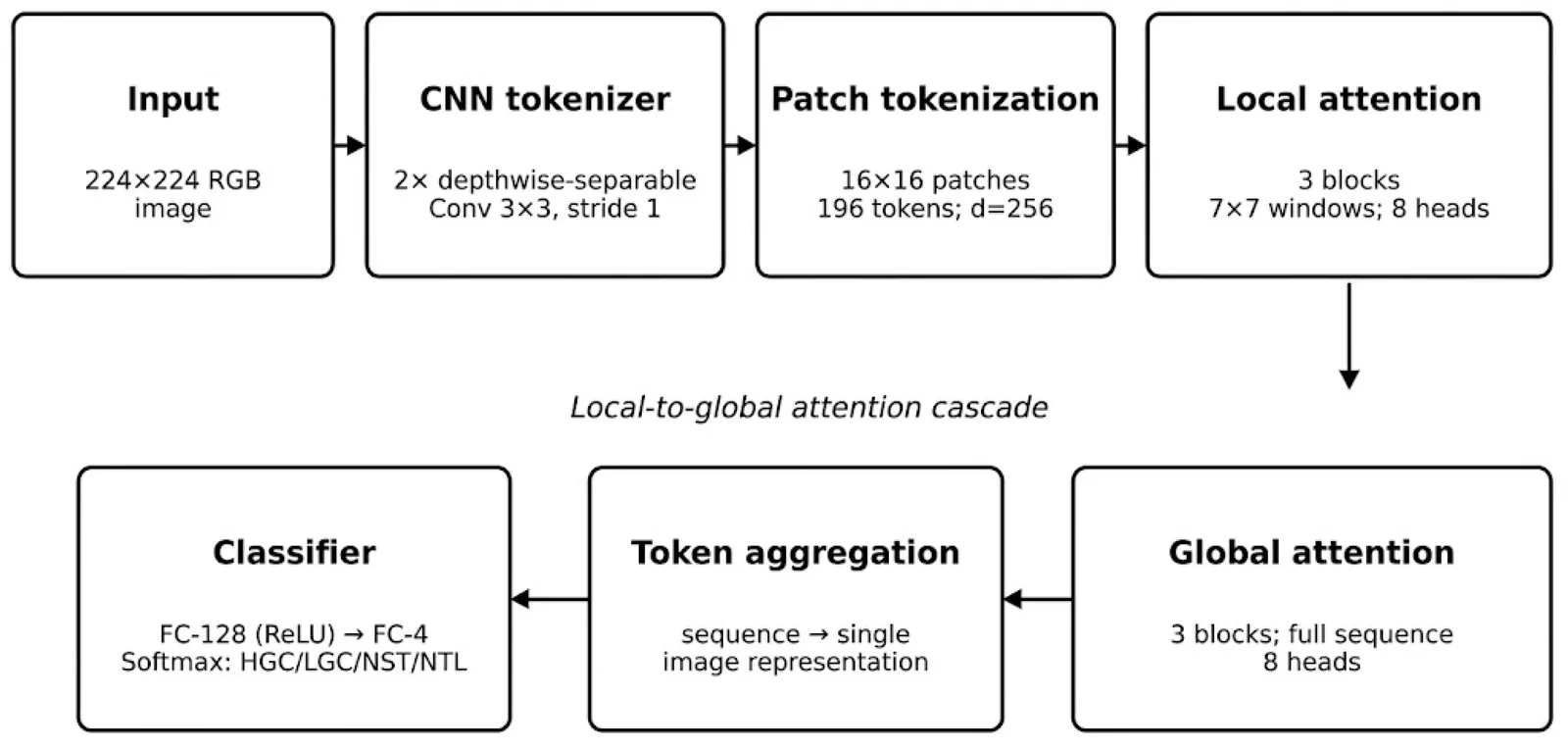
Schematic overview of the proposed LGH-Transformer architecture. The CNN tokenizer preserves local image structure before tokenization; local attention models short-range tissue relationships, whereas global full-sequence attention integrates long-range contextual information prior to four-class classification.

By combining convolutional tokenization with local and global attention, the LGH-Transformer provides a testable hybrid architecture for exploratory internal evaluation. Algorithm 1, Figure 10, and Table 8 describe the implemented architecture: two sequential depthwise-separable convolutional tokenizer layers; 16 × 16 tokenization yielding 196 tokens at d_model = 256; three local-attention blocks followed by three global-attention blocks; and an FC-128 (ReLU) → FC-4 → Softmax classification head. The reported ~18.2 M parameters and ~4.1 GFLOPs refer to this specification.

The proposed LGH-Transformer architecture is specified consistently in Algorithm 1 and Table 8; Algorithm 1 is the procedural representation of the same component sequence and hyperparameters summarized in Table 8.
**Algorithm 1.** LGH-Transformer Forward Architecture (Aligned with Table 8)Input: X ∈ ℝ^(324 × 224 × 3), RGB cystoscopic image Output: ŷ ∈ ℝ^4^, four-class probability vector**Canonical Architecture Constraints**two depthwise-separable convolutional tokenizer layers16 × 16 tokenization (196 tokens)three local-attention blocks followed sequentially by three global-attention blocksFC-128 (ReLU) → Softmax classifier[CLS]-token formulation and interleaved local/global loop are not used↓1.**Layer 1: Depthwise-separable Convolution** F_1_ = DSConv_1_(X), kernel = 3 × 3, stride = 1↓2.**Layer 2: Patch Tokenization** T^(0)^ = Tokenize_16×16_(DSConv_2_(F_1_)) → 196 image tokens↓3.**Loop 1: for l = 1 to 3 do** Local Attention Block T^(l)^ = LA_l_(T^(l−1)^; window = 7 × 7, heads = 8)↓4.**Loop 2: for g = 1 to 3 do** Global Attention Block G^(0)^ = T^(3)^, G^(g)^ = GA^g^(G^(g−1)^; full sequence, heads = 8)↓5.**Aggregate token sequence to a single d_model-dimensional image representation** z = Aggregate(G^(3)^), z ∈ ℝ^(d_model)↓6.**Linear Classifier 1: ReLU(FC_128_(z))** h = ReLU(W_1_z + b_1_), h ∈ ℝ^128^↓7.**Linear Classifier 2: Softmax(FC_4_(h))** o = W_2_h + b_2_, ŷ_i_ = exp(o_i_)/Σ_j=1_^4^ exp(o_j_)↓8.**Return ŷ** ŷ ∈ ℝ^4^, Σ_i=1_^4^ ŷ_i_ = 1

To provide a clearer block-level representation of the proposed architecture, Figure 10 provides a dedicated schematic distinct from the procedural representation in Algorithm 1. The schematic explicitly shows the progression from the two-layer depthwise-separable CNN tokenizer to 16 × 16 patch tokenization (196 tokens; d_model = 256), three 7 × 7 local-attention blocks, three full-sequence global-attention blocks, token aggregation, and the FC-128 (ReLU) → FC-4 → Softmax classifier. For direct visual comparison with the benchmark models, simplified architecture schematics for the remaining 17 models are provided in Supplementary Figure S1.

**Figure 11:**
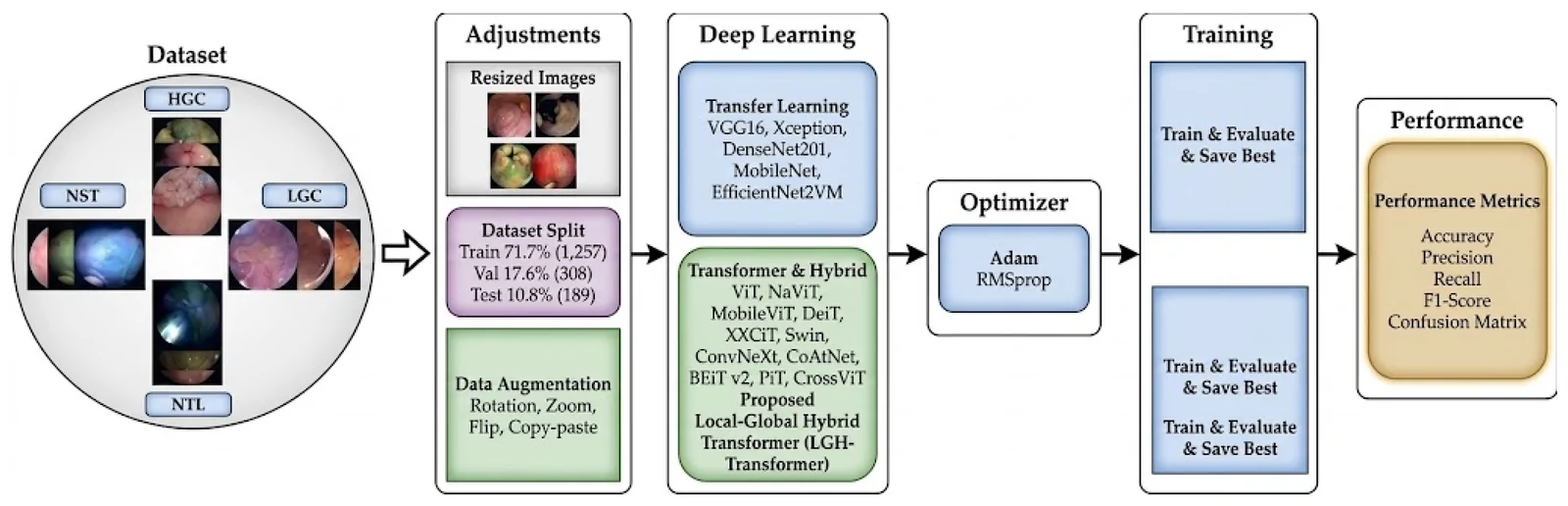
Workflow diagram of the 18 evaluated architectures.

A schematic of the methodological framework is shown in Figure 11. The workflow comprises the 18 evaluated architectures: five CNN transfer-learning models and thirteen Transformer-based/hybrid models.

### 4.4. Experimental Setup

The four tissue classes were not equally represented in the dataset, with the NTL class being the least frequent. Patient-level splitting was completed before augmentation. No class weighting, random oversampling, or undersampling was applied during model training. Instead, data augmentation was restricted to the training subset to increase visual variability, including for the minority NTL class. The validation and held-out test sets were left unchanged and retained their naturally occurring class distributions. This design avoided altering the class composition of the evaluation sets and ensured that reported performance reflected the original distribution of the held-out patients.

Eighteen image-classification architectures were evaluated. The CNN group comprised VGG16, Xception, DenseNet201, MobileNet, and EfficientNetV2M. The Transformer-based and hybrid group comprised ViT Tiny Patch 16, ViT Base Patch 16, MobileViT, DeiT, XCiT Medium 24 P16, Swin Base, ConvNeXt Base, CoAtNet, BEiT v2, PiT-S224, CrossViT Small, and the proposed LGH-Transformer.

All models used a common classification head consisting of a fully connected layer with 128 neurons and ReLU activation followed by a four-class softmax output. Adam and RMSprop were evaluated systematically for each architecture. The initial learning rate was optimized separately for each model, whereas the broader grid search over batch size, dropout, and weight decay was restricted to the proposed LGH-Transformer and the best-performing transfer-learning models (MobileNet and Xception). Thus, optimizer comparisons were performed within each architecture, but not all hyperparameters were identically re-optimized across every architecture.

The final Softmax layer generates continuous probability distributions across the four tissue categories (HGC, LGC, NST, and NTL) in addition to the final predicted class label. These probability outputs represent model confidence scores and were not interpreted as direct clinical probabilities.

Each pretrained Transformer/hybrid benchmark backbone was fine-tuned independently with Adam and RMSprop, and the proposed LGH-Transformer was trained according to its study-specific implementation under both optimizers. For every run, the study-specific four-class head was trainable and the best validation checkpoint was retained for final held-out evaluation. This wording distinguishes the pretrained benchmark models from the custom LGH-Transformer without introducing undocumented checkpoint or layer-freezing details.

For all models, the initial learning rate was optimized separately. Early stopping was applied to transfer-learning models when validation loss failed to improve for ten consecutive epochs (patience = 10).

During preprocessing, all images were resized to fit the 224 × 224 input dimensions of the models. Additionally, data augmentation techniques such as rotation, zoom, and horizontal flipping were used to improve model generalization. The experiments were performed using an NVIDIA GeForce RTX 3090 GPU with 24 GB VRAM and the PyTorch (Versions 1.0.0 v2.13.0) deep-learning library (Section 4.7.3). Models were trained for 50 epochs. Model performance was evaluated using accuracy, loss, macro-precision, macro-recall, macro-F1 score, ROC-AUC, and confusion matrices.

### 4.5. Evaluation Metrics

All models were evaluated under the publicly released held-out test framework. The annotation-defined test set contains 189 frames with class totals HGC = 74, LGC = 53, NST = 37, and NTL = 25, and each confusion matrix displayed in Figure 1, Figure 2, Figure 3 and Figure 4 uses these same row totals. For each displayed matrix, accuracy was recalculated as the sum of the diagonal entries divided by 189 and compared with the corresponding table value; all four comparisons match exactly to four decimal places. No class weighting, oversampling, undersampling, or augmentation was applied to the test data. Because overall accuracy can be influenced by class imbalance, performance was additionally characterized using macro-averaged precision, recall, F1-score, confusion matrices, and class-specific sensitivity, specificity, PPV, and NPV. The test set was used only for final evaluation and was not involved in model selection, hyperparameter tuning, or balancing procedures.

Accuracy was defined as the total number of correct predictions divided by the number of held-out frames. For each class k, one-versus-rest precision was calculated as TP_k/(TP_k + FP_k), recall as TP_k/(TP_k + FN_k), and F1_k as 2 × Precision_k × Recall_k/(Precision_k + Recall_k).

ROC-AUC was calculated from continuous class-probability outputs as a descriptive metric; no formal inferential between-model ROC comparisons were performed.

The Precision, Recall, and F1-Score values reported in Table 1, Table 2, Table 3 and Table 4 are macro-averaged metrics: Macro Precision = (1/K)Σ_k Precision_k, Macro Recall = (1/K)Σ_k Recall_k, and Macro F1 = (1/K)Σ_k F1_k, with K = 4 classes (HGC, LGC, NST, and NTL). Therefore, the reported Precision, Recall, and F1-Score values are neither micro-averaged nor prevalence-weighted averages. Confusion matrices, accuracy, and the macro-averaged confusion-derived metrics were generated from the same experimental prediction records so that the reported values are directly traceable to the underlying integer prediction counts.

For interpretation of repeated numerical values, accuracy, precision, recall, and F1-score are functions of the final discrete class predictions. Consequently, two different architectures or optimizer settings can yield the same rounded values when their discrete predictions produce the same aggregate class-count patterns. All performance values are reported to four decimal places, which can further increase the appearance of numerical ties.

Furthermore, a confusion matrix was generated to facilitate a more detailed analysis of prediction performance. This matrix displays the number of correct and incorrect predictions for each class, revealing which classes the model performs well on and which it struggles with. In addition to these aggregate metrics, per-class Sensitivity (True Positive Rate), Specificity (True Negative Rate), Positive Predictive Value (PPV), and Negative Predictive Value (NPV) were calculated for the prespecified LGH-Transformer–Adam configuration using a one-versus-rest scheme. These measures are reported descriptively as point estimates only. Confidence intervals and frame-level inferential significance tests were not used because multiple correlated frames originated from the same patients.

### 4.6. Computational Complexity Analysis

In order to evaluate the computational characteristics of the models under study, a computational complexity analysis was conducted for all architectures. Table 5 lists the complete set of 18 evaluated architectures. Parameter numbers and FLOPs for established architectures were taken from their respective original publications and official implementations where available. For the proposed LGH-Transformer, the reported approximately 18.2 million trainable parameters and 4.1 GFLOPs correspond to the architecture defined in Table 8 and Algorithm 1 and were obtained from the implemented PyTorch (Versions 1.0.0 v2.13.0) architecture using torch info.

### 4.7. Patient-Wise Data Split, Hyperparameter Tuning, and Reproducibility

#### 4.7.1. Patient-Wise Data Split

The publicly released annotation-defined split contains 1754 frames: 1257 training, 308 validation, and 189 held-out test frames. The corresponding class distributions are training: HGC = 309, LGC = 482, NST = 379, NTL = 87; validation: HGC = 86, LGC = 112, NST = 88, NTL = 22; and test: HGC = 74, LGC = 53, NST = 37, NTL = 25. The held-out test subset contains five patients. All augmentation was restricted to the training subset after the fixed split was established.

Frame distribution across the publicly released 1257/308/189 training, validation, and held-out test subsets. The class counts shown in Table 9 are taken from the dataset annotation file and define the split used throughout this study.

#### 4.7.2. Hyperparameter Tuning

Hyperparameter tuning was conducted without using the held-out test set. A structured grid search was performed for the proposed LGH-Transformer and the best-performing transfer-learning models (MobileNet and Xception) over learning rate {1 × 10^−5^, 5 × 10^−5^, 1 × 10^−4^, 5 × 10^−4^}, batch size {16, 32, 64}, dropout {0.1, 0.2, 0.3}, and weight decay {0, 1 × 10^−4^, 1 × 10^−3^}. Candidate configurations were fitted using training-patient data and selected according to validation-set performance; the held-out test patients were not used for model selection, early stopping decisions, or hyperparameter optimization. Data augmentation was applied only to the training subset after patient-level splitting. No separate handcrafted feature-selection stage was used. Preprocessing operations requiring estimated parameters were derived from the training data only; fixed image scaling was applied identically to the validation and test images. Early stopping used validation loss with a patience of 10 epochs.

The most suitable hyperparameter settings for the LGH-Transformer were learning rate = 1 × 10^−4^ (Adam), batch size = 32, dropout = 0.1, weight decay = 1 × 10^−4^. As to MobileNet (Adam), it was found that the best configuration was: learning rate = 5 × 10^−5^, batch size = 32, dropout = 0.1.

For the secondary stability analysis, the final selected LGH-Transformer configuration was applied across a five-fold stratified patient-wise cross-validation procedure with the random seed fixed at 42. Patient identity was used as the grouping unit; thus, all frames from a given patient remained within a single fold. The exact fold-level class distributions and experimentally observed fold-specific ROC-AUC values are reported in Supplementary Tables S1 and S2. This cross-validation analysis is reported separately from the primary fixed public split and was not used to replace the held-out test results.

#### 4.7.3. Reproducibility, Prediction-Level Audit, and Automatic Output Generation

These materials comprise: (1) source code for data loading, model construction, training, checkpoint selection, evaluation, ablation experiments, and figure/table generation; (2) the fixed public 1257/308/189 split manifest; (3) the archived five-fold patient-wise cross-validation assignment/class-distribution record and fold-specific LGH-Transformer ROC-AUC outputs summarized in Supplementary Tables S1 and S2; (4) per-sample prediction files for the primary evaluated model-optimizer and ablation runs; (5) the run-level random-seed record; and (6) the software/hardware environment record. For every evaluated sample in the primary held-out analysis, the prediction files store the sample identifier, patient identifier where available, true class, predicted class, and class-probability outputs for HGC, LGC, NST, and NTL. These files correspond to the results reported in the primary tables and figures.

Model-initialization provenance is recorded as part of the reproducibility metadata. VGG16, Xception, DenseNet201, MobileNet, EfficientNetV2M, ViT Tiny Patch 16, ViT Base Patch 16, MobileViT, DeiT, XCiT Medium 24 P16, Swin Base, ConvNeXt Base, CoAtNet, BEiT v2, PiT-S224, and CrossViT Small are identified as pretrained/fine-tuned benchmark models, whereas LGH-Transformer is identified as the study-specific custom architecture. Exact checkpoint identifiers and layer-freezing/unfreezing settings, where applicable, are implementation details stored with the source-code/configuration records.

Prediction-level traceability is defined at the per-sample level as: sample_ID, patient_ID where available, true_label, predicted_label, P(HGC), P(LGC), P(NST), and P(NTL). The predicted label is the class with the highest stored probability. The supplementary prediction file therefore permits direct regeneration of the confusion matrices, accuracy, macro-precision, macro-recall, macro-F1, and ROC-AUC without manual transcription.

Run metadata for reproducibility is reported as follows: the random seed was fixed at 42 for Python, NumPy, and PyTorch. The software/hardware environment was Python 3.10.12, NumPy 1.26.4, scikit-learn 1.4.2, PyTorch 2.2.2, CUDA 12.1, cuDNN 8.9.7, and an NVIDIA GeForce RTX 3090 GPU with 24 GB VRAM (NVIDIA driver 535.104.05).

## 5. Conclusions

This study compared 18 deep learning architectures for multiclass bladder tissue classification from cystoscopic images under a common patient-wise evaluation framework using the fixed public 1257/308/189 split. The highest held-out accuracy, 187/189 (0.9894), was shared by seven model–optimizer configurations, including the proposed LGH-Transformer–RMSprop; because these results differ by only one to two frames on a 189-frame test set containing five patients, they constitute a top-accuracy tier rather than evidence of unique superiority. The distinguishing result for the LGH-Transformer is efficiency: it reached this tier with approximately 18.2 M parameters and 4.1 GFLOPs, the smallest footprint among the top-tier models. Component-wise ablation showed modest accuracy decreases after removal of the CNN tokenizer (0.0106), local attention (0.0053), and global attention (0.0053), and a decrease of 0.0212 when all three were removed, supporting complementary component contributions within this internal split. The evidence level remains exploratory internal validation: these findings should not be interpreted as evidence of clinical utility, external generalizability, or statistical superiority. Independent multicenter validation with adequate patient numbers, patient-level analysis, and repeated-seed evaluation is required before deployment can be considered.

## Figures and Tables

**Table 1:** Transfer Learning Model: Performance with Adam.

Model	Optimizer	Accuracy (189-Frame Test)	Macro Precision	Macro Recall	Macro F1-Score	ROC-AUC
VGG16	Adam	0.8942	0.9122	0.8623	0.8744	0.9341
Xception	Adam	0.9735	0.9707	0.9755	0.9720	0.9886
DenseNet201	Adam	0.9735	0.9812	0.9779	0.9792	0.9874
MobileNet	Adam	0.9788	0.9776	0.9751	0.9762	0.9911
EfficientNetV2M	Adam	0.8466	0.8736	0.8176	0.8185	0.9027

**Table 2:** Transfer Learning Model: Performance with RMSprop.

Model	Optimizer	Accuracy (189-Frame Test)	Macro Precision	Macro Recall	Macro F1-Score	ROC-AUC
VGG16	RMSprop	0.8730	0.8889	0.8528	0.8522	0.9192
Xception	RMSprop	0.9788	0.9790	0.9772	0.9778	0.9962
DenseNet201	RMSprop	0.9894	0.9905	0.9905	0.9905	0.9951
MobileNet	RMSprop	0.9735	0.9777	0.9678	0.9718	0.9885
EfficientNetV2M	RMSprop	0.8942	0.9042	0.8784	0.8774	0.9416

**Table 3:** Transformer Model: Performance with Adam.

Model	Optimizer	Accuracy (189-Frame Test)	Macro Precision	Macro Recall	Macro F1-Score	ROC-AUC
ViT (baseline)	Adam	0.8783	0.8363	0.8338	0.8340	0.9302
MobileViT	Adam	0.9683	0.9618	0.9604	0.9605	0.9833
DeiT	Adam	0.9788	0.9682	0.9594	0.9634	0.9904
XCiT Medium 24 P16	Adam	0.9894	0.9906	0.9667	0.9773	0.9963
Swin Base	Adam	0.9841	0.9860	0.9500	0.9651	0.9954
ConvNeXt Base	Adam	0.9894	0.9906	0.9667	0.9773	0.9952
CoAtNet	Adam	0.9894	0.9906	0.9667	0.9773	0.9948
ViT Tiny Patch 16	Adam	0.9524	0.9596	0.8996	0.9207	0.9782
BEiT v2	Adam	0.8783	0.9012	0.8002	0.8290	0.9414
PiT-S224	Adam	0.8942	0.8693	0.8163	0.8328	0.9494
ViT Base Patch 16	Adam	0.9683	0.9581	0.9251	0.9390	0.9857
CrossViT Small	Adam	0.9841	0.9871	0.9500	0.9657	0.9940
Proposed LGH-Transformer	Adam	0.9841	0.9840	0.9785	0.9811	0.9973

**Table 4:** Transformer Model: Performance with RMSprop.

Model	Optimizer	Accuracy (189-Frame Test)	Macro Precision	Macro Recall	Macro F1-Score	ROC-AUC
ViT (baseline)	RMSprop	0.8519	0.8368	0.8339	0.8338	0.9148
MobileViT	RMSprop	0.9471	0.9422	0.9408	0.9408	0.9744
DeiT	RMSprop	0.9841	0.9738	0.9735	0.9736	0.9928
XCiT Medium 24 P16	RMSprop	0.9735	0.9535	0.9520	0.9523	0.9879
Swin Base	RMSprop	0.9841	0.9860	0.9500	0.9651	0.9954
ConvNeXt Base	RMSprop	0.9894	0.9906	0.9667	0.9773	0.9946
CoAtNet	RMSprop	0.9894	0.9906	0.9667	0.9773	0.9942
ViT Tiny Patch 16	RMSprop	0.9524	0.9596	0.8996	0.9207	0.9779
BEiT v2	RMSprop	0.8783	0.9012	0.8002	0.8290	0.9409
PiT-S224	RMSprop	0.8942	0.8693	0.8163	0.8328	0.9489
ViT Base Patch 16	RMSprop	0.9683	0.9581	0.9251	0.9390	0.9852
CrossViT Small	RMSprop	0.9841	0.9871	0.9500	0.9657	0.9936
Proposed LGH-Transformer	RMSprop	0.9894	0.9901	0.9800	0.9846	0.9959

**Table 5:** Computational complexity of all evaluated models (224 × 224 input).

Model	Parameters (M)	FLOPs (G)	Held-Out Accuracy (Best Optimizer Configuration; *n* = 189)
VGG16	138.4	15.5	0.8942
Xception	22.9	8.4	0.9735
DenseNet201	20.0	4.3	0.9735
MobileNet	4.2	0.57	0.9788
EfficientNetV2M	54.1	24.0	0.8466
ViT Tiny Patch 16	5.7	1.3	0.9524
ViT Base Patch 16	86.6	17.6	0.9683
MobileViT	5.6	2.0	0.9683
DeiT	86.6	17.6	0.9788
XCiT Medium 24 P16	84.4	16.2	0.9894
Swin Base	87.8	15.4	0.9841
ConvNeXt Base	88.6	15.4	0.9894
CoAtNet	42.0	8.4	0.9894
BEiT v2	86.5	17.6	0.8783
PiT-S224	23.5	2.9	0.8942
CrossViT Small	26.9	5.6	0.9841
Proposed LGH-Transformer	18.2 M	4.1	0.9841

**Table 6:** Descriptive per-class Sensitivity, Specificity, PPV, and NPV for the proposed LGH-Transformer (Adam optimizer), recalculated from the 189-frame confusion matrix in Figure 3. One-versus-rest scheme; point estimates only.

Class	Sensitivity	Specificity	PPV	NPV
**HGC**	1.0000	1.0000	1.0000	1.0000
**LGC**	0.9811	0.9853	0.9630	0.9926
**NST**	0.9730	0.9934	0.9730	0.9934
**NTL**	0.9600	1.0000	1.0000	0.9939
**Macro Avg.**	0.9785	0.9947	0.9840	0.9950

**Table 7:** Component-wise ablation analysis of the proposed LGH-Transformer.

Variant	CNN Tokenizer	Local Attention	Global Attention	Accuracy (*n* = 189)	Macro Precision	Macro Recall	Macro F1	ROC-AUC	Accuracy Drop
Full LGH-Transformer	Included	Included	Included	0.9841	0.9840	0.9785	0.9811	0.9973	0.0000
Without CNN tokenizer	Removed	Included	Included	0.9735	0.9768	0.9650	0.9704	0.9918	0.0106
Without local attention	Included	Removed	Included	0.9788	0.9817	0.9702	0.9756	0.9940	0.0053
Without global attention	Included	Included	Removed	0.9788	0.9811	0.9689	0.9747	0.9931	0.0053
No tokenizer + no local + no global	Removed	Removed	Removed	0.9630	0.9680	0.9542	0.9603	0.9859	0.0212

**Table 8:** Proposed LGH-Transformer Architecture Summary.

Component/Hyper-Parameter	Specification
Input resolution	224 × 224 px (3-channel RGB)
Patch size (tokenization)	16 × 16 px → 196 tokens per image
CNN tokenizer	2 sequential Depthwise Separable Conv layers (stride 1, kernel 3 × 3)
Embedding dimension (d_model)	256
Local attention blocks	3 sequential blocks—window size 7 × 7, 8 heads, MLP ratio 4×
Global attention blocks	3 sequential blocks after the local blocks—full sequence, 8 heads, MLP ratio 4×
Attention heads (per block)	8
Feed-forward (MLP) hidden dim	1024 (4 × d_model)
Dropout rate	0.1 (attention + MLP)
Classification head	FC-128 (ReLU) → FC-4 → Softmax (4 classes)
Total trainable parameters	~18.2 M
FLOPs (224 × 224 input)	~4.1 GFLOPs
Optimizer yielding highest held-out accuracy	RMSprop (0.9894)
Training epochs	50
Attention order	CNN tokenizer → 196 tokens → 3 local-attention blocks → 3 global-attention blocks → FC-128 (ReLU) → Softmax

**Table 9:** Patient-Wise Data Split, Hyperparameter Tuning, and Reproducibility.

Subset	Patients/Metadata	HGC	LGC	NST	NTL	Total Frames
Training	14 patients; fixed public split	309	482	379	87	1257
Validation	4 patients; fixed public split	86	112	88	22	308
Test	5 patients; fixed public split	74	53	37	25	189
Total	Fixed public split	469	647	504	134	1754

## Data Availability

The image dataset and annotation file used in this study are publicly available from the Endoscopic Bladder Tissue Classification Dataset repository (Zenodo record 7741476; see Ref. [23]). The primary analysis uses the fixed public 1257/308/189 split recorded in the released annotation file.

## Supplementary Materials

The following supporting information can be downloaded at: https://www.mdpi.com/article/10.3390/ijms27198929/s1.

## Abbreviations

Abbreviation	Full Term
AI	Artificial Intelligence
BEiT	Bidirectional Encoder Representation from Image Transformers
BLC	Blue Light Cystoscopy
CIS	Carcinoma In Situ
CNN	Convolutional Neural Network
DeiT	Data-efficient Image Transformer
FLOPs	Floating-Point Operations
HGC	High-Grade Cancer
LGC	Low-Grade Cancer
LGH-Transformer	Local–Global Hybrid Transformer
MLP	Multi-Layer Perceptron
MRI	Magnetic Resonance Imaging
NBI	Narrow Band Imaging
NMIBC	Non-Muscle-Invasive Bladder Cancer
NPV	Negative Predictive Value
NST	Non-Suspicious Tissue
NTL	No Tumor Lesion
PiT	Pooling-based Vision Transformer
PPV	Positive Predictive Value
ReLU	Rectified Linear Unit
TURBT	Transurethral Resection of Bladder Tumor
VGG	Visual Geometry Group
ViT	Vision Transformer
WHO	World Health Organization
WLC	White Light Cystoscopy
WLI	White Light Imaging
XCiT	Cross-Covariance Image Transformer
