# Supplementary material for: LGH-Transformer: A Hybrid CNN–Transformer Model for Multi-Class Bladder Tissue Classification from Cystoscopic Images
Source: Int J Mol Sci. 2026 Oct 8;27(19):8929. doi: 10.3390/ijms27198929 (PMC13666353; doi:10.3390/ijms27198929)
Supplement: Supplementary file 1 [file ijms-27-08929-s001.zip › ijms-4563777-supplementary.pdf]

Supplementary Figure S1. Block-Level Schematic Comparison of the 17 Benchmark Models

Models are grouped by architectural family to make the main information-flow differences easier to compare with the proposed LGH-Transformer.

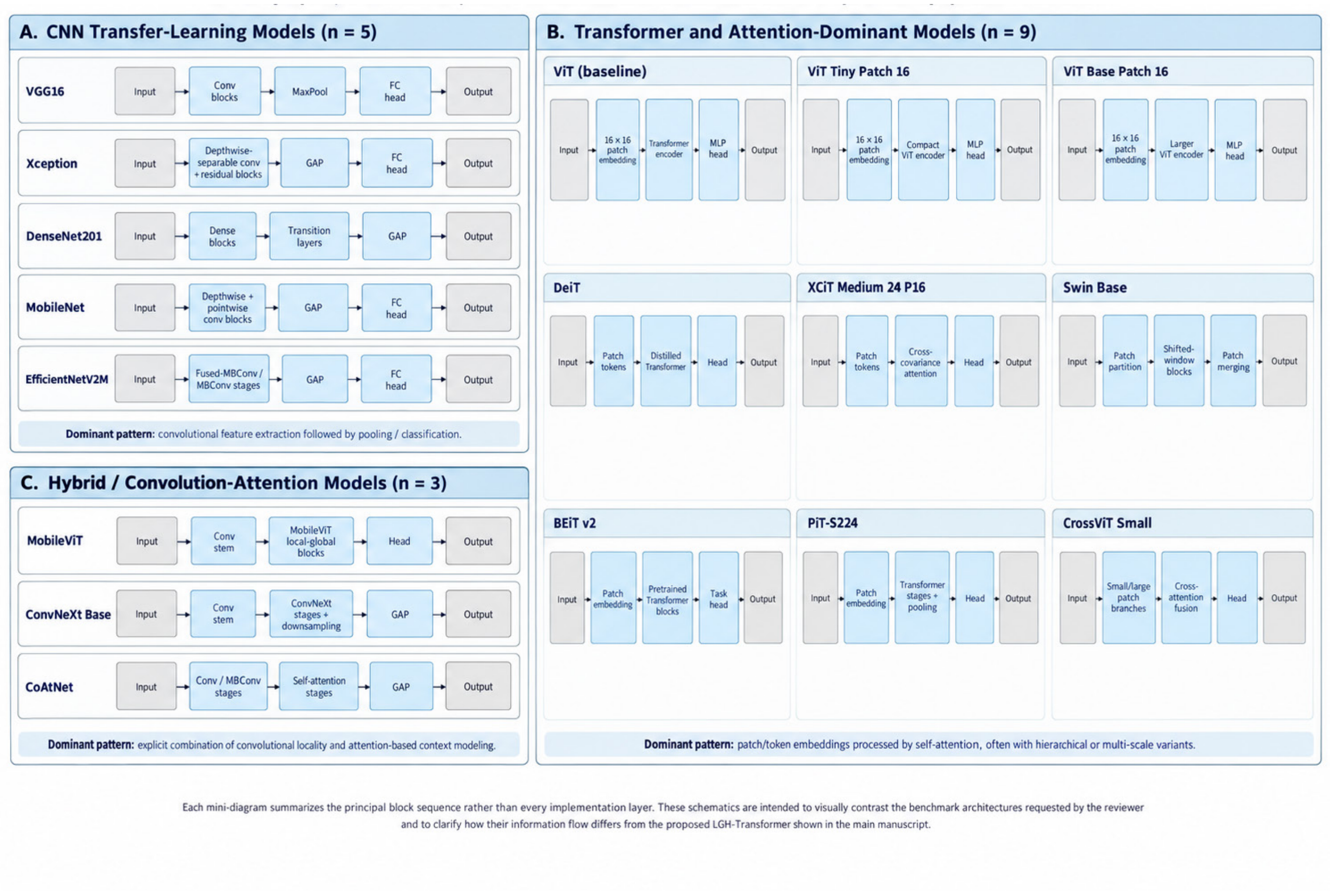

Caption: Simplified block-level schematics of the 17 benchmark architectures evaluated alongside the proposed LGH-Transformer. Models are grouped into CNN transfer-learning backbones, Transformer / attention-dominant architectures, and hybrid convolution-attention models. Each mini-diagram summarizes the dominant sequence of processing blocks to facilitate visual comparison of information flow, tokenization strategy, and attention usage across the evaluated architectures.

representations of the benchmark CNN, Transformer, and hybrid architectures to facilitate visual comparison.

Supplementary Table S1. Experimental fold-level class distribution in the five-fold stratified patient-wise cross-validation analysis.

Fold	HGC	LGC	NST	NTL	Total
Fold 1	94	130	101	27	352
Fold 2	93	129	101	27	350
Fold 3	94	130	101	27	352
Fold 4	94	129	101	27	351
Fold 5	94	129	100	26	349
Total	469	647	504	134	1754

Supplementary Table S2. Experimental fold-specific ROC-AUC values of the proposed LGH-Transformer.

Fold	LGH-Transformer Adam	LGH-Transformer RMSprop
Fold 1	0.9898	0.9979
Fold 2	0.9921	0.9991
Fold 3	0.9910	0.9988
Fold 4	0.9930	0.9994
Fold 5	0.9916	0.9993
Mean	0.9915	0.9989
Standard deviation	0.0012	0.0006

Note: Fold-specific confusion-derived accuracy, sensitivity, specificity, PPV, and NPV values were not available in the archived cross-validation summary and therefore were not retrospectively estimated.
